# Ambient air pollution associated with incidence and dynamic progression of type 2 diabetes: a trajectory analysis of a population-based cohort

**DOI:** 10.1186/s12916-022-02573-0

**Published:** 2022-10-31

**Authors:** Yinglin Wu, Shiyu Zhang, Samantha E. Qian, Miao Cai, Haitao Li, Chongjian Wang, Hongtao Zou, Lan Chen, Michael G. Vaughn, Stephen Edward McMillin, Hualiang Lin

**Affiliations:** 1 Department of Epidemiology, School of Public Health, Sun Yat-sen University, Guangzhou, 510080 China; 2 College for Public Health & Social Justice, Saint Louis University, Saint Louis, MO 63104 USA; 3 Department of Social Medicine and Health Service Management, Health Science Center, Shenzhen University, Shenzhen, 518060 China; 4 Department of Epidemiology and Biostatistics, College of Public Health, Zhengzhou University, Zhengzhou, 450001 China; 5 School of Social Work, College for Public Health & Social Justice, Saint Louis University, Saint Louis, MO 63103 USA

**Keywords:** Air pollution, Type 2 diabetes, Diabetes complication, Diabetes mortality, Multi-state model

## Abstract

**Background:**

Though the association between air pollution and incident type 2 diabetes (T2D) has been well documented, evidence on the association with development of subsequent diabetes complications and post-diabetes mortality is scarce. We investigate whether air pollution is associated with different progressions and outcomes of T2D.

**Methods:**

Based on the UK Biobank, 398,993 participants free of diabetes and diabetes-related events at recruitment were included in this analysis. Exposures to particulate matter with a diameter ≤ 10 μm (PM_10_), PM_2.5_, nitrogen oxides (NO_x_), and NO_2_ for each transition stage were estimated at each participant’s residential addresses using data from the UK’s Department for Environment, Food and Rural Affairs. The outcomes were incident T2D, diabetes complications (diabetic kidney disease, diabetic eye disease, diabetic neuropathy disease, peripheral vascular disease, cardiovascular events, and metabolic events), all-cause mortality, and cause-specific mortality. Multi-state model was used to analyze the impact of air pollution on different progressions of T2D. Cumulative transition probabilities of different stages of T2D under different air pollution levels were estimated.

**Results:**

During the 12-year follow-up, 13,393 incident T2D patients were identified, of whom, 3791 developed diabetes complications and 1335 died. We observed that air pollution was associated with different progression stages of T2D with different magnitudes. In a multivariate model, the hazard ratios [95% confidence interval (CI)] per interquartile range elevation in PM_2.5_ were 1.63 (1.59, 1.67) and 1.08 (1.03, 1.13) for transitions from healthy to T2D and from T2D to complications, and 1.50 (1.47, 1.53), 1.49 (1.36, 1.64), and 1.54 (1.35, 1.76) for mortality risk from baseline, T2D, and diabetes complications, respectively. Generally, we observed stronger estimates of four air pollutants on transition from baseline to incident T2D than those on other transitions. Moreover, we found significant associations between four air pollutants and mortality risk due to cancer and cardiovascular diseases from T2D or diabetes complications. The cumulative transition probability was generally higher among those with higher levels of air pollution exposure.

**Conclusions:**

This study indicates that ambient air pollution exposure may contribute to increased risk of incidence and progressions of T2D, but to diverse extents for different progressions.

**Supplementary Information:**

The online version contains supplementary material available at 10.1186/s12916-022-02573-0.

## Background

Type 2 diabetes (T2D), caused by insulin resistance and pancreatic beta cell dysfunction, accounts for approximately 90% of all diabetes cases [1, 2]. Approximately 463 million people worldwide suffered from diabetes in 2019, and the prevalence continues to rise [3]. More than half of patients will develop any one of complications, and these diabetes-related complications represent the major causes of disability and mortality due to T2D [4, 5]. Owing to the advances in the treatment of T2D, many individuals will survive longer with these conditions, leading to an increased economic burden in the presence of diabetes complications [4, 6].

Existing evidence supports an association between the risk of T2D and ambient air pollution exposure, especially for fine particulate matter pollution (PM_2.5_) and nitrogen dioxide (NO_2_) [7, 8]. The biological mechanisms underlying this association include immune activation, endoplasmic reticulum stress, central nervous system inflammation, and oxidative stress [9, 10]. Additionally, previous studies have found that ambient air pollution was associated with some complications of diabetes, including diabetic retinopathy, incident cardiovascular diseases, and chronic kidney diseases [11–13]. However, it remained unknown whether ambient air pollution was associated with the dynamic progression of T2D, such as from baseline to incident T2D, further to diabetes complications, and subsequent to death. Based on the previous studies, it is reasonable to hypothesize that ambient air pollution could increase the risk of diabetes complications and subsequent mortality, in other words, exposure to higher levels of ambient air pollution could be associated with an increased risk of different progressions of T2D. The investigation on the effect of risk factors in T2D trajectory would be important for specific interventions at different stages of T2D.

We utilized a multi-state model to assess the association between air pollution exposure and different progressions of T2D. We further predicted the transition probabilities of different stages for participants exposed to different levels of air pollution. The findings from this analysis will add to the evidence to prioritize action to reduce ambient air pollution and optimize prevention and management strategies of T2D.

## Methods

### Study design

The UK Biobank is a large population-based cohort study. Details of the UK Biobank have been described previously [14]. In brief, the cohort included more than 500,000 participants aged 37–73 years at baseline (2006–2010) in 22 sites across England, Scotland, and Wales, and each participant was followed up. All participants completed a baseline survey, including socioeconomic factors, lifestyle factors, and the history of medication.

In this analysis, we included 398,993 participants who were free of diabetes and any diabetes-related events (cardiovascular diseases, diabetes eye diseases, diabetes kidney diseases, and diabetes neuropathy diseases) at baseline (Additional file 1: Fig. S1). Participants with occurrences of the diabetes-related events before the diagnosis of T2D were excluded (Additional file 1: Fig. S1). Participants with missing exposure data were also excluded, whose residential addresses were geocoded outside the range of assessment (Additional file 1: Fig. S1). The prevalent cases of diabetes and any diabetes-related events at baseline were assessed based on self-reported information, medication history, and hospital inpatient records (supplemental material).

This study was approved by the North West Multicenter Research Ethics Committee, and informed consent was obtained from all participants.

### Environmental exposure assessment

The mean annual concentrations of particulate matter with a diameter ≤10 μm (PM_10_), PM_2.5_, nitrogen oxides (NO_x_), and NO_2_ were collected from the UK’s Department for Environment, Food and Rural Affairs (DEFRA), a platform providing high-resolution near-surface air pollution data in the UK from 2002 to 2020 [15]. Annual concentration maps of various air pollutants were modeled on 1 km × 1 km grid using an air dispersion model based on different sources from the National Atmospheric Emissions Inventory, a combination of measurement data for secondary inorganic aerosol, and models for sources including resuspension of dust. These concentrations were calibrated using measured concentrations taken from background sites in Defra's Automatic Urban and Rural Network [15].

**Fig. 1:**
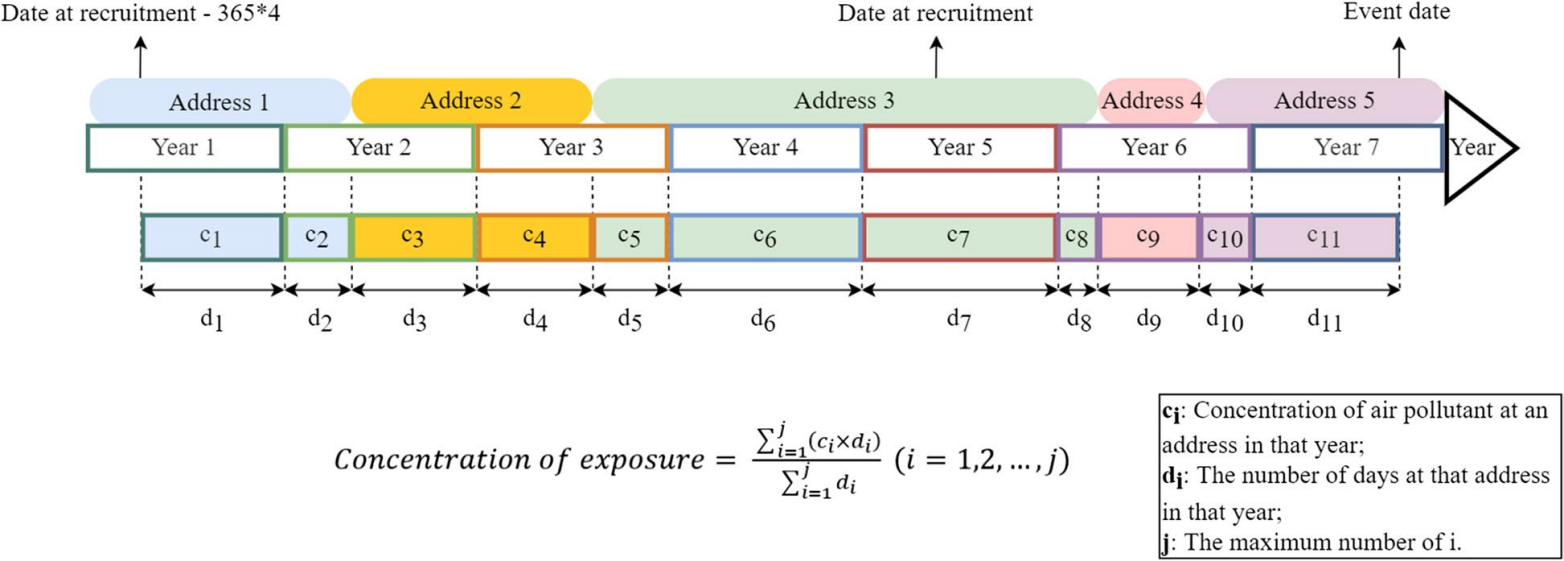
The air pollution exposure estimation strategy. The colorful, rounded rectangles represent the participant’s address. The rectangles with colorful borders represent each year during the follow-up period. The color-filled rectangles with colorful borders represent the concentration of air pollutants at that address in that current year. The fill-in color corresponds to that of the address, and the border color matches that of the current year

Given the exposures at baseline may not accurately represent the air pollution exposure over the long follow-up period, we estimated exposures to four air pollutants from the date of 4 years before recruitment to the dates when any outcome occurred. To assess the air pollutant exposures for each participant, we collected participants’ residential address history including the dates that participants lived in each location. The annual mean air pollution concentration for each residential location was estimated by the following processes. First, the annual mean concentration of air pollutants of the current year was assigned to the participant by 1km × 1km grid cells in which they resided. Second, we counted the number of days of residence at that address in the calendar year. Third, we calculated average levels of the air pollutant exposures for each participant by weighting the time spent at each residential address. The formula can be specified as:

$$\mathrm{Concentration}\ \mathrm{of}\ \mathrm{air}\ \mathrm{pollution}\ \mathrm{exposure}=\frac{\sum_{i=1}^j\left({c}_i\times {d}_i\right)}{\sum_{i=1}^j{d}_i}\ \left(i=1,2,\dots, j\right),$$

where *c*_*i*_ is the annual mean concentration at an address in that year, *d*_*i*_ is the days at that address in the calendar year, and *j* is the number of combinations of different addresses and corresponding days in a calendar year. The exposure estimation strategy was presented in Fig. 1.

### Follow-up and ascertainment of outcomes

All the participants were followed up since the date when the participants consented to join the UK Biobank study, and the end date was either death, loss to follow-up, or the last date of follow-up on 31 March 2021, whichever occurred first.

The key outcomes of interest in this study were the incident T2D, diabetes complications events, and mortality. During the follow-up, T2D and diabetes complications were identified through gathering information from hospital admissions, primary care, and death records, provided by the UK National Health Service. Diabetes complications were defined as the first occurrence of any diabetes complications events after the diagnosis of incident T2D [16]. Diabetes complications in this study included diabetic eye diseases, diabetic kidney diseases, diabetic neuropathy diseases, cardiovascular diseases (CVD), peripheral vascular diseases, and metabolic events. All-cause mortality and cause-specific mortality were obtained from the national death registry. Cause-specific mortality included cancer, CVD, and respiratory diseases, which accounted for 62.1%, 10.2%, and 6.2% of the overall deaths, respectively. The detailed information on codes and data fields of the key outcomes was presented in the Additional file 1: Details of outcomes.

### Covariates

We considered a series of important covariates that have been shown to be associated with either T2D or ambient air pollution in this analysis, including demographics, socioeconomic factors, lifestyle, and comorbidities [1, 2, 8]. Demographic, socioeconomic factors and lifestyle factors included age at recruitment (continuous variable), sex, ethnicity (white and non-white), living area (urban and rural), smoking status (never, previous, and current), healthy diet (including intake of fish, meat, vegetables, fruit, and alcohol consumption), and physical activity (low, moderate, and high). Obesity was evaluated by a body mass index higher than 30 kg/m^2^. Comorbidities were assessed based on self-reported data, medication history, and hospital inpatient data, including hypertension, high cholesterol, and cancer. Details of the assessment of covariates are displayed in Additional file 1: Table S1. The missing data on covariates were imputed with multivariate imputation via a chained equation.

### Statistical analysis

The correlation between air pollutant exposures was assessed by Spearman’s correlation coefficients. We utilized a multi-state regression model to estimate the hazard ratio (HR) and 95% confidence interval (CI) for the associations between per interquartile range (IQR) increase in air pollutants and progression trajectories of T2D from healthy to incident T2D, further to complication, and ultimately to death. We also assessed the associations between air pollution and cause-specific mortality risk from baseline, T2D, and diabetes complications. The multi-state model is an extension of the competing risk model to assess the impact of certain exposures on different stages of disease progression [17, 18], and has been applied in several studies on the associations between lifestyle and the risk of cardiometabolic multimorbidity [19, 20].

**Fig. 2:**
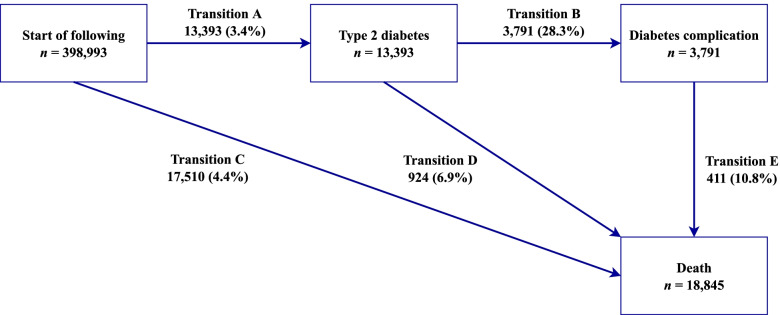
Transitions from baseline to T2D, diabetes complications, and all-cause death. Diabetes complications included diabetic eye diseases, diabetic kidney diseases, diabetic neuropathy diseases, cardiovascular diseases, peripheral vascular diseases, and metabolic events. State-specific number of events was reported in boxes, and the transition-specific number of events and percentages (within brackets) were reported on arrows

In this study, five transition phases were considered based on the natural history of T2D (Fig. 2) [2]: (A) baseline to T2D (*n* = 13,393); (B) T2D to any one of diabetes complications (*n* = 3791); (C) baseline to death without T2D (*n* = 17,510); (D) T2D to death from any cause (*n* = 924); (E) diabetes complications to death from any cause (*n* = 411). Moreover, transition patterns from T2D to cause-specific mortality were presented in Additional file 1: Fig. S2. For participants who experienced multiple states on the same date (*n* = 2075), the entry date of the first state was calculated as the entry date of the later state minus the median interval time of each stage in this study (598 days for transition B; 564 days for transition D; 485 days for transition E). For example, for participants diagnosed with diabetes and related complications on the same day, the date of onset of T2D was equal to the date of complications minus 598 days.

We constructed 3 models to examine the associations between ambient air pollutants and five transitions of T2D trajectory. In the basic model, we adjusted for age, sex, and ethnicity. In model 2, we additionally adjusted for the potential confounders listed above. As obesity and chronic disease history could be on the causal pathway between air pollution and T2D, we excluded obesity and chronic diseases in model 3. We then used model 3 as the primary model for the subsequent analyses to avoid the potential bias.

We used restricted cubic splines with three knots to estimate deviations from linearity in exposure-response of the association between air pollutants and different stages of T2D trajectory and used likelihood ratio tests to examine the nonlinear associations.

We predicted the cumulative probabilities of the later state from the prior state during the follow-up among participants living with different levels of air pollution. Cumulative predicted probabilities were calculated for men and women aged 55 years old, with the maximized distribution of the observed adjusted factors (ethnicity, residential area, healthy diets, physical activity, smoking status, family history of diabetes).

### Sensitivity analysis

To assess the robustness of the results, we further conducted several sensitivity analyses: (1) 2-pollutant models for each of the 4 air pollutants by including 2 air pollutants in the same model. Because PM_10_ and PM_2.5_ were strongly correlated (Spearman’s correlation coefficient > 0.9), and NO_x_ and NO_2_ were also strongly correlated, we did not include them in the same model [21]; (2) further adjustment for traffic noise as traffic noise may be the potential confounder of the association between air pollution and incidence of T2D [22, 23]; (3) using different time intervals (1 day, 1 year, and 3 years) to calculate the entry date of the previous state for participants entering different states on the same day; (4) restricting the mortality due to T2D or diabetes complications; (5) excluding participants without complete data of covariates; and (6) excluding the participants who were diagnosed with T2D or diabetes complications on the same date.

### Stratified analysis

We further conducted several analyses stratified by age, residential area, smoking status, physical activity, and healthy diet. We calculated *p* values using likelihood ratio tests comparing models with and without multiplicative interaction terms.

All the statistical analyses were performed using R software (version 4.0.3). The missing data were imputed using the ‘mice’ package. The multi-state model was run using the “mstate” package [17]. Results with a 2-sided *p* value < 0.05 were considered statistically significant.

## Results

### Descriptive results

A total of 398,993 participants included in the study were younger than those excluded and more likely to be female and white (Additional file 1: Table S2). The mean age of participants included was 55.49 years (SD 8.06 years). Over half were female (57.1%) and most participants were white (94.4%). During a mean follow-up of 12.0 (SD = 1.5) years [4,768,126 person-years (PYs)], 13,393 (28.1/10,000 PYs) participants experienced T2D (Fig. 2). Among these participants, 3791 (555.2/10,000 PYs) developed complications, and 924 (135.3/10,000 PYs) died without experiencing complications. Of the 3791 cases with diabetes complications, 411 died with a crude mortality rate of 237.2 per 10,000 PYs. The primary cause of death was cancer followed by CVD and respiratory disease (Additional file 1: Fig. S2).

Table 1 presents the baseline characteristics of participants by the top and bottom quartiles of exposure to PM_2.5_ and NO_2_ in the transition from baseline to T2D. Participants with higher exposure to ambient air pollution were younger and more likely to be living in urban area and current smokers. The mean exposures to PM_10_, PM_2.5_, NO_x_, and NO_2_ in the whole stage were 15.04, 10.01, 27.27, and 18.23 μg/m^3^, respectively (Additional file 1: Table S3). These four exposures were highly positively correlated with pairwise correlation coefficients higher than 0.78 (*p* < 0.05, Additional file 1: Fig. S3).

**Table 1:** Characteristics of 398,993 participants

	Overall (***n*** = 398,993)	PM_**2.5**_ (μg/m^**3**^)		NO_**2**_ (μg/m^**3**^)	
		Q1: 1.69–8.74 (***n*** = 99,752)	Q4: 11.05–18.70 (***n*** = 99,747)	Q1: 2.86–14.17 (***n*** = 99,750)	Q4: 21.25–57.92 (***n*** = 99,748)
Age [years, mean (SD)]	55.49 (8.06)	55.86 (7.93)	55.20 (8.24)	56.18 (7.85)	54.75 (8.25)
Sex (%)					
Male	171,055 (42.9)	41,922 (42.0)	42,957 (43.1)	42,212 (42.3)	43,543 (43.7)
Female	227,938 (57.1)	57,830 (58.0)	56,790 (56.9)	57,538 (57.7)	56,205 (56.3)
Ethnicity (%)					
White	376,743 (94.4)	98,181 (98.4)	85,835 (86.1)	98,301 (98.5)	85,601 (85.8)
Non-white	22,250 (5.6)	1571 (1.6)	13,912 (13.9)	1449 (1.5)	14,147 (14.2)
Residential area (%)					
Urban	343,011 (86.0)	78,809 (79.0)	97,350 (97.6)	58,586 (58.7)	98,935 (99.2)
Rural	55,982 (14.0)	20,943 (21.0)	2397 (2.4)	41,164 (41.3)	813 (0.8)
Smoking status (%)					
Never	227,700 (57.1)	59,195 (59.3)	54,593 (54.7)	59,787 (59.9)	53,265 (53.4)
Previous	131,114 (32.9)	31,749 (31.8)	33,344 (33.4)	32,536 (32.6)	33,135 (33.2)
Current	40,179 (10.1)	8808 (8.8)	11,810 (11.8)	7427 (7.4)	13,348 (13.4)
Obese (%)					
Yes	110,765 (27.8)	20,755 (20.8)	20,420 (20.5)	19,748 (19.8)	21,832 (21.9)
No	387,917 (97.2)	78,997 (79.2)	79,327 (79.5)	80,002 (80.2)	77,916 (78.1)
Physical activity (%)					
Low	72,398 (18.1)	17,204 (17.2)	17,645 (17.7)	17,443 (17.5)	18,077 (18.1)
Moderate	164,071 (41.1)	40,976 (41.1)	42,436 (42.5)	40,854 (41.0)	41,698 (41.8)
High	162,524 (40.7)	41,572 (41.7)	39,666 (39.8)	41,453 (41.6)	39,973 (40.1)
Healthy diet (%)					
Yes	284,828 (71.4)	70,418 (70.6)	72,492 (72.7)	72,301 (72.5)	71,609 (71.8)
No	114,165 (28.6)	29,334 (29.4)	27,255 (27.3)	27,449 (27.5)	28,139 (28.2)
Family history of diabetes (%)					
Yes	83,680 (21.0)	19,848 (19.9)	21,617 (21.7)	19,748 (19.8)	22,202 (22.3)
No	315,313 (79.0)	79,904 (80.1)	78,130 (78.3)	80,002 (80.2)	77,546 (77.7)
Chronic diseases (%)					
High cholesterol	41,841 (10.5)	9998 (10.0)	11,129 (11.2)	9515 (9.5)	11,518 (11.5)
Hypertension	161,003 (40.4)	39,475 (39.6)	38,540 (38.6)	40,945 (41.0)	38,778 (38.9)
Cancer	42,225 (10.6)	10,669 (10.7)	10,516 (10.5)	10,900 (10.9)	10,248 (10.3)

### Association between air pollution and different progressions of type 2 diabetes

The associations between air pollution and risk of different progressions of T2D were presented in Table 2. In the basic model, all four air pollutants were significantly associated with an increased risk of dynamic progressions of T2D. The results in model 2 were stable when we additionally adjusted for lifestyle factors, obesity, and history of chronic diseases.

**Table 2:** Associations between air pollution and risk of five progressions of T2D using the multi-state model

Transition	Cases	PM_**10**_	PM_**2.5**_	NO_**x**_	NO_**2**_
**Basic model**					
Baseline → T2D	13,393	1.66 (1.63, 1.70)	1.63 (1.60, 1.67)	1.39 (1.37, 1.41)	1.47 (1.44, 1.49)
T2D → complication	3791	1.14 (1.09, 1.19)	1.09 (1.05, 1.14)	1.11 (1.07, 1.15)	1.16 (1.11, 1.20)
Baseline → death	17,510	1.54 (1.51, 1.57)	1.52 (1.49, 1.55)	1.33 (1.31, 1.35)	1.39 (1.37, 1.41)
T2D → death	924	1.43 (1.31, 1.56)	1.51 (1.38, 1.65)	1.22 (1.14, 1.31)	1.27 (1.18, 1.37)
Complication → death	411	1.49 (1.31, 1.71)	1.57 (1.38, 1.80)	1.39 (1.25, 1.55)	1.44 (1.28, 1.62)
**Model 2**					
Baseline → T2D	13,393	1.67 (1.63, 1.71)	1.64 (1.61, 1.68)	1.40 (1.38, 1.42)	1.49 (1.46, 1.52)
T2D → complication	3791	1.13 (1.08, 1.18)	1.08 (1.03, 1.13)	1.10 (1.06, 1.15)	1.15 (1.11, 1.20)
Baseline → death	17,510	1.51 (1.48, 1.54)	1.50 (1.47, 1.53)	1.32 (1.30, 1.34)	1.39 (1.37, 1.41)
T2D → death	924	1.42 (1.29, 1.55)	1.50 (1.37, 1.64)	1.22 (1.14, 1.31)	1.28 (1.18, 1.38)
Complication → death	411	1.45 (1.27, 1.65)	1.53 (1.34, 1.74)	1.35 (1.21, 1.51)	1.41 (1.24, 1.59)
**Model 3**					
Baseline → T2D	13,393	1.66 (1.62, 1.69)	1.63 (1.59, 1.67)	1.39 (1.37, 1.42)	1.49 (1.46, 1.51)
T2D → complication	3791	1.13 (1.08, 1.18)	1.08 (1.03, 1.13)	1.10 (1.06, 1.15)	1.15 (1.11, 1.20)
Baseline → death	17,510	1.51 (1.48, 1.54)	1.50 (1.47, 1.53)	1.32 (1.30, 1.34)	1.39 (1.36, 1.41)
T2D → death	924	1.41 (1.29, 1.54)	1.49 (1.36, 1.64)	1.22 (1.13, 1.31)	1.27 (1.17, 1.37)
Complication → death	411	1.46 (1.28, 1.67)	1.54 (1.35, 1.76)	1.36 (1.22, 1.52)	1.42 (1.25, 1.60)

HRs (95% CI) are results for per IQR increase from multi-state models. IQR increase was 3.25 μg/m^3^ for PM_10_, 2.31 μg/m^3^ for PM_2.5_, 12.43 μg/m^3^ for NO_x_, and 7.08 μg/m^3^ for NO_2_. All values are statistically significant (*p* < 0.05)

Basic model was adjusted for age, sex, and ethnicity

Model 2 was based on a basic model and additionally adjusted for residential area, smoking status, healthy diet, physical activity, family history of diabetes, obesity, history of hypertension, high cholesterol, and cancer

Model 3 was adjusted for age, sex, ethnicity, residential area, smoking status, healthy diet, physical activity, and family history of diabetes

*Abbreviations*: *CI* confidence interval, *HR* hazard ratio, *IQR* interquartile

In model 3, results persisted and remained robust (Table 2). For example, the HRs (95% CI) per IQR elevation (2.31 μg/m^3^) in PM_2.5_ were 1.63 (1.59, 1.67) and 1.08 (1.03, 1.13) for transitions from healthy to T2D and from T2D to complications, and 1.50 (1.47, 1.53), 1.49 (1.36, 1.64), and 1.54 (1.35, 1.76) for mortality risk from baseline, T2D, and diabetes complications, respectively. The associations of four air pollutants on incident T2D were generally stronger than those on other transitions, and the HRs (95% CI) per IQR increase in PM_10_, NO_x_, and NO_2_ exposure were 1.66 (1.62, 1.69), 1.39 (1.37, 1.42), and 1.49 (1.46, 1.51), respectively. Generally, the estimates for transition from diabetes complications to death were slightly stronger than that from T2D directly to death. For example, the HR (95% CI) of mortality from diabetes complications per IQR (7.08 μg/m^3^) increase in NO_2_ exposure was 1.42 (1.25, 1.60), and the estimate was 1.27 (1.17, 1.37) for mortality from T2D.

The positive associations between four exposures and dynamic progressions of T2D were observed to be monotonic across the range of ambient air pollution exposures, except for the association between particulate matter and transition from T2D to diabetes complications (Additional file 1: Fig. S4).

The associations between air pollution and risk of cause-specific mortality were shown in Additional file 1: Table S4. Four air pollution exposures were positively associated with increased risks of cancer, CVD, and respiratory diseases mortality from T2D or diabetes complication. The associations between air pollution and the risk of cancer mortality from T2D were stronger than those for mortality due to other causes. The estimates for transition from diabetes complications to CVD mortality were stronger than those for other cause-specific mortality.

### Cumulative transition probability of type 2 diabetes trajectories

**Fig. 3:**
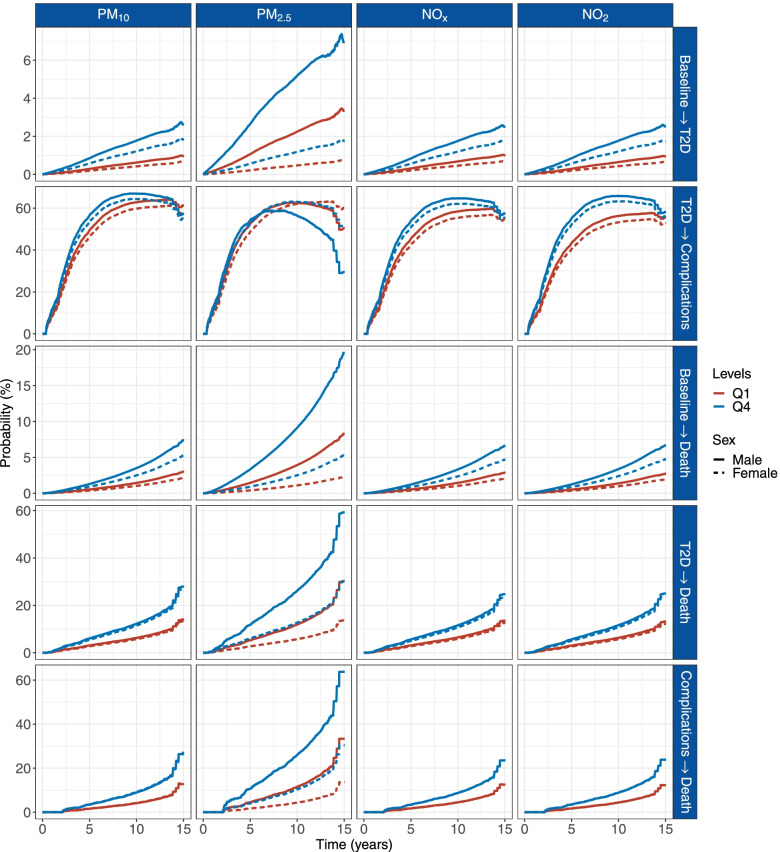
Cumulative transition probabilities of T2D for participants exposed to different levels of air pollution. Computed for 55-year-old men (continuous) and women (dotted) for values of air pollution of quartile 1 and quartile 4. Quartile cutoffs were 2.92–13.23 μg/m^3^ and 16.48–28.48 μg/m^3^ for PM_10_, 1.69–8.74 μg/m^3^ and 11.05–18.70 μg/m^3^ for PM_2.5_, 3.75–19.79 μg/m^3^ and 32.22–111.01 μg/m^3^ for NO_x_, and 2.86–14.17 μg/m^3^ and 21.25–57.92 μg/m^3^ for NO_2_, respectively. The model was adjusted for age, sex, ethnicity, living area, smoking status, healthy diet, physical activity, and family history of diabetes. Abbreviation: T2D, type 2 diabetes

Figure 3 shows the cumulative transition probabilities of different progressions for participants aged 55 years old during the follow-up period. The transition probabilities of development of T2D, diabetes complications, and post-diabetes mortality were generally higher in participants living in a higher level of air pollution. For example, the cumulative probabilities for T2D in healthy people at 14.7 years of follow-up were 2.7% and 1.9% for men and women, respectively, living with the top quartile of PM_10_, and were 1.0% and 0.7% for men and women, respectively, living with the lowest quartile. Comparing to those living in the lowest quartile of NO_2_, the cumulative probabilities for diabetes complications in patients with T2D living in the top quartile exposure at 14.9 years of follow-up increased by approximately 3% (58.2% in Q4 vs. 55.6% in Q1 for men and 56.2% in Q4 vs. 52.9% in Q1 for women). The difference in cumulative mortality between the highest quartile of NO_x_ and the lowest quartile in patients with diabetes complications at 14.5 years of follow-up was approximately 11% (23.5% in Q4 vs. 12.5% in Q1 for men; 23.6% in Q4 vs. 12.6% in Q1 for women).

### Sensitivity analyses

In the 2-pollutant model, the associations between PM_10_ and PM_2.5_ and different transitions of T2D remained significant and stable, except for the associations with transition from T2D to diabetes complications. The associations of NO_x_ and NO_2_ with progressions of T2D remained significant, except for those with mortality risk from T2D or diabetes complications (Additional file 1: Table S5). In the model including traffic noise, we observed stable associations between the four air pollutants and transitions from baseline to T2D, further to diabetes complication, and subsequent to death (Additional file 1: Table S6). The associations in sensitivity analyses using different time intervals did not significantly change (Additional file 1: Table S7). In sensitivity analyses excluding deaths not due to T2D or diabetes-related events, the estimates decreased and associations of four exposures with transition from T2D to death became insignificant (Additional file 1: Table S8). The results after excluding participants with missing data were similar to those from our main multivariable models (Additional file 1: Table S9). When excluding the participants who were diagnosed with T2D or diabetes complications on the same date, the estimates increased for transitions from baseline to T2D and subsequently to diabetes complications but decreased for transitions to death from T2D or diabetes complications (Additional file 1: Table S10).

### Stratified analyses

There was a suggestion of effect modification by residential area across different exposures (Additional file 1: Fig. S5–S8). Stronger estimates on increased risk of T2D were found in those living in a rural area (*p*-for-interaction <0.001). For example, the HRs (95% CI) for incident T2D in participants living in a rural area and an urban area were 1.97 (1.81, 2.14) and 1.38 (1.36, 1.40) per IQR (12.43 μg/m^3^) increment in NO_x_, respectively. We also observed stronger associations between air pollution and transition from T2D to diabetes complications in those living in a rural area (*p*-for-interaction 0.061 for PM_10_, <0.001 for PM_2.5_, NO_x_, and NO_2_).

## Discussion

This study provided some novel findings by showing the positive associations between four air pollutants (PM_10_, PM_2.5_, NO_x_, and NO_2_) and different transition stages from healthy to T2D, to diabetes complications, and then to death. We also quantified the cumulative probabilities of five transitions and found that participants exposed to higher levels of air pollution may have an increased risk of adverse diabetic outcomes, particularly the risk of diabetes complications and diabetic mortality. Furthermore, there was a consistent evidence of effect modification by residential areas across different exposures.

Several systematic reviews have summarized the association between air pollution exposure and the risk of T2D [7, 24], and these studies support a positive association. For example, one recent meta-analysis reviewed 86 studies and reported the HRs (95% CI) of incident T2D associated with each 10 μg/m^3^ increment in PM_2.5_ and PM_10_ were 1.10 (1.04, 1.17) and 1.11 (1.00, 1.22), respectively [7]. Consistent with these studies, our study found the positive associations of PM_10_, PM_2.5_, NO_x_, and NO_2_ with transition from baseline to T2D. The difference in estimates may be due to ambient air pollution exposures among the UK Biobank participants being much lower than those in previous studies conducted in developing countries. Furthermore, variations in pollution characteristics and susceptability differences among different populations cannot be excluded.

Despite several studies that have explored the associations of ambient T2D and heart failure with ambient air pollution using the UK Biobank [25, 26], we attempted to use this study to uncover the associations between air pollution and dynamic progression of T2D, rather than just one disease state. In the current study, we observed the significantly adverse associations between air pollution and risk of incident T2D, diabetes complications, and mortality from T2D or diabetes complications. The estimates of incident T2D risk in our analyses were stronger than those reported in previous study, possibly due to the mulit-state model adjusted for competing risk from mortality [25].

Although air pollution is linked to T2D, the association with the dynamic transitions is not clear. Previous studies reported a significantly positive association between ambient air pollutants (PM_2.5_ and NO_2_) and risk of cardiovascular disease risk among patients with diabetes, diabetic retinopathy, microalbuminuria, and hospitalization of acute complications [13, 27–29], whereas other studies did not [30, 31]. In the present study, high exposure to PM_10_, PM_2.5_, NO_2_, and NO_x_ was found to be positively associated with risk of any one of diabetes complications from T2D, and the associations were relatively weaker than other transitions.

In line with previous studies, our results showed a positive association between ambient air pollutants and the transition from baseline to death [32]. Previously studies conducted in different countries found a positive association between long-term exposure to air pollution and diabetes mortality [33–36]. Beyond the evidence mentioned above, we hypothesized that air pollution were associated with mortality from T2D and diabetes complications. In the present study, significant positive associations was observed between ambient air pollution exposure and transition stages for all cause mortality and cause-specific mortality (cancer and CVD mortality) from T2D or diabetes complications. Generally, the estimates for the transition from T2D to death were slightly weaker than those for the transition from diabetes complications to death and were attenuated when excluding the mortality, not due to T2D or diabetes complications. This may support evidence that the association of air pollution with diabetes mortality may be explained in large part by the fact that air exposures might increase risk of fatal events in patients with diabetes complications. The differences in our results may be due to several methodological issues. First, previous studies explored the transition from healthy to diabetes mortality. As such, this does not reflect whether air pollution effects were different on the dynamic transition. Second, although diabetes has been found to be a major cause of mortality, most patients with diabetes may die mainly from diabetes complications [37]. Previous studies collected diabetes-associated mortality according to the ICD code for diabetes, which may omit potential premature mortality due to diabetes complications. Lastly, the competing risk from death may lead to a violation of the independent censoring assumption and could alter the risk estimates.

There are some studies elucidating the biological mechanisms of relationship between air pollution and T2D. Both epidemiological studies and animal experiments have shown that exposure to air pollution can induce vascular insulin resistance and metabolic disturbances by elevating pulmonary oxidative stress [38, 39]. Exposure to PM_2.5_ might involve hypothalamic inflammation via an active sympathetic nervous system [40]. Long-term exposures to PM_10_ and NO_2_ were also associated with elevated levels of systematic subclinical inflammation, and adipokines [10]. Moreover, recent studies also found that air pollution was associated with chronic hyperglycemia, dyslipidemia, and blood pressure variation in diabetes patients [41–43]. These factors may trigger the release of inflammatory factors, generate reactive oxygen species, exacerbate abnormalities in endothelial function, and increase the risk of both micro- and macrovascular diseases in diabetes [44–46].

In this study, we observed some notable evidence for effect modification. Our results found stronger associations between air pollution exposure and incidence of T2D among participants residing in a rural area, consistent with previous findings [47]. This may be associated with some specific community types, such as healthy food access and the construction of primary health care.

Although ambient air pollution is considered as a leading risk factor for diabetic morbidity and mortality [48], this cannot reflect the association on the dynamic progression. Our findings provide evidence that ambient air pollution, including particulate matter and nitrogen oxides, is associated with both the incidence and the subsequent progressions of T2D. This suggests ambient air pollution may contribute to both the long-term progression of T2D and the mortality risk of existing disease. In addition, the estimates of air pollution were generally stronger on transition stage from baseline to T2D than in other transition stages, and the associations with diabetes mortality were also noteworthy. Our results inform that more attention should be paid not only to the primary prevention of T2D, but also to the prevention of air pollution after the diagnosis of T2D, so as to potentially mitigate the development of diabetes complications and reduce premature mortality.

The major strengths of our study were a large population-based cohort and the detailed information on socioeconomic, behavioral, and clinical profile data in the UK Biobank, which enabled us to structure a multi-state model of T2D development and to control for potential confounding factors. Compared with traditional Cox regression models, we used the multi-state model to distinguish the impacts of air pollution on the five transition phases. Thus, we were able to detect the sensitive stages of diabetes development and how progression can be affected by the pollution. A series of sensitivity analyses confirmed the robustness of the results.

Our study had some limitations. The participants in the UK Biobank were healthy-volunteer. Second, the outcome identification relied on the ICD-10 codes, and some of the complications are not specific to T2D. The identification of diabetes complications may be open to potential misclassification. Third, some participants were simultaneously diagnosed with diabetes and its complications, which can also lead to outcome misclassification. We then excluded the participants diagnosed with T2D and diabetes complications on the same date, and the associations between air pollution and different progressions of T2D remained significant and positive. Lastly, some information (e.g., work environment) was unavailable from the UK Biobank which would result in exposure misclassifications.

## Conclusions

In conclusion, air pollution was differentially positively associated with the progression trajectories of T2D. Reducing air pollution exposure prior to T2D and diabetes complications may also contribute to a favorable longevity of lifetime by reducing the risk of premature mortality. These findings provide the evidence for adhering to public health recommendations to control air pollution.

## Supplementary Information

**Additional file 1: **Details of outcomes. **Table S1.** The UDI, definition and measurements of covariates. **Table S2.** Characteristics of the participants included or excluded in the study. **Table S3.** Distributions of the annual average exposures among 398,993 participants. **Table S4.** Associations between air pollution and risk of cause-specific mortality. **Table S5.** Results of 2-pollutant models. **Table S6.** Results of sensitivity analyses in the model including traffic noise (*n* = 393,515). **Table S7.** Results of sensitivity analyses using different time intervals. **Table S8.** Results of sensitivity analyses after excluding deaths not from diabetes or diabetes complications (*n* = 380,437). **Table S9.** Results of sensitivity analyses using complete data (*n* = 318,019). **Table S10.** Results excluding the participants diagnosed with T2D and complications on the same date (*n* = 396,961). **Figure S1.** Flowchart of participants included in this study. **Figure S2.** Transitions from baseline to T2D, diabetes complications, and cause-specific mortality. **Figure S3.** Spearman’s correlation coefficients between air pollutant exposures. **Figure S4.** Exposure-response associations between air pollution exposure and different transitions of T2D. **Figure S5.** Effect modifications of the association between PM_10_ and five transitions of T2D. **Figure S6.** Effect modifications of the association between PM_2.5_ and five transitions of T2D. **Figure S7.** Effect modifications of the association between NO_x_ and five transitions of T2D. **Figure S8.** Effect modifications of the association between NO_2_ and five transitions of T2D.

## Data Availability

The datasets are available upon reasonable request to the Access Management System (AMS) through the UK Biobank website (https://www.ukbiobank.ac.uk/enable-your-research/apply-for-access). The air pollution exposure data are available from DEFRA (https://uk-air.defra.gov.uk/data/pcm-data).

## Abbreviations

CVD: Cardiovascular diseases
DEFRA: Department for Environment, Food and Rural Affairs
NO_2_: Nitrogen dioxide
NO_x_: Nitrogen oxide
PM_10_: Particulate matter with a diameter ≤10 μm
PM_2.5_: Particulate matter with a diameter ≤2.5 μm
T2D: Type 2 diabetes
