# Supplementary material for: Ambient air pollution associated with incidence and dynamic progression of type 2 diabetes: a trajectory analysis of a population-based cohort
Source: BMC Med. 2022 Oct 31;20:375. doi: 10.1186/s12916-022-02573-0 (PMC9620670; doi:10.1186/s12916-022-02573-0)
Supplement: Supplementary file 1 — Additional file 1: Details of outcomes. Table S1. The UDI, definition and measurements of covariates. Table S2. Characteristics of the participants included or excluded in the study. Table S3. Distributions of the annual average exposures among 398,993 participants. Table S4. Associations between air pollution and risk of cause-specific mortality. Table S5. Results of 2-pollutant models. Table S6. Results of sensitivity analyses in the model including traffic noise (n = 393,515). Table S7. Results of sensitivity analyses using different time intervals. Table S8. Results of sensitivity analyses after excluding deaths not from diabetes or diabetes complications (n = 380,437). Table S9. Results of sensitivity analyses using complete data (n = 318,019). Table S10. Results excluding the participants diagnosed with T2D and complications on the same date (n = 396,961). Figure S1. Flowchart of participants included in this study. Figure S2. Transitions from baseline to T2D, diabetes complications, and cause-specific mortality. Figure S3. Spearman’s correlation coefficients between air pollutant exposures. Figure S4. Exposure-response associations between air pollution exposure and different transitions of T2D. Figure S5. Effect modifications of the association between PM10 and five transitions of T2D. Figure S6. Effect modifications of the association between PM2.5 and five transitions of T2D. Figure S7. Effect modifications of the association between NOx and five transitions of T2D. Figure S8. Effect modifications of the association between NO2 and five transitions of T2D. [file 12916_2022_2573_MOESM1_ESM.docx]

**Supplemental Material**

**Ambient air pollution associated with incidence and dynamic progression of type 2 diabetes: a trajectory analysis of a population-based cohort**

Yinglin Wu, Shiyu Zhang, Samantha E. Qian, Miao Cai, Haitao Li, Chongjian Wang, Hongtao Zou, Lan Chen, Michael G. Vaughn, Stephen Edward McMillin, Hualiang Lin

Table of Contents

[Details of outcomes 3](#_Toc112353231)

[Table S1. The UDI, definition and measurements of covariates. 5](#_Toc112353232)

[Table S2. Characteristics of the participants included or excluded in the study. 7](#_Toc112353233)

[Table S3. Distributions of the annual average exposures among 398,993 participants. 8](#_Toc112353234)

[Table S4. Associations between air pollution and risk of cause-specific mortality. 9](#_Toc112353235)

[Table S5. Results of 2-pollutant models. 10](#_Toc112353236)

[Table S6. Results of sensitivity analyses in the model including traffic noise (*n* = 393,515). 11](#_Toc112353237)

[Table S7. Results of sensitivity analyses using different time intervals. 12](#_Toc112353238)

[Table S8. Results of sensitivity analyses after excluding deaths not from diabetes or diabetes complication (*n* = 380,437). 13](#_Toc112353239)

[Table S9. Results of sensitivity analyses using complete data (*n* = 318,019). 14](#_Toc112353240)

[Table S10. Results excluding the participants diagnosed with T2D and complications on the same date (*n* = 396,961). 15](#_Toc112353241)

[Figure S1. Flowchart of participants included in this study. 16](#_Toc112353242)

[Figure S2. Transitions from baseline to T2D, diabetes complication, and cause-specific mortality. 17](#_Toc112353243)

[Figure S3. Spearman’s correlation coefficients between air pollutant exposures. 18](#_Toc112353244)

[Figure S4. Exposure-response associations between air pollution exposure and different transitions of T2D. 19](#_Toc112353245)

[Figure S5. Effect modifications of the association between PM_10_ and five transitions of T2D. 20](#_Toc112353246)

[Figure S6. Effect modifications of the association between PM_2.5_ and five transitions of T2D. 21](#_Toc112353247)

[Figure S7. Effect modifications of the association between NO_x_ and five transitions of T2D. 22](#_Toc112353248)

[Figure S8. Effect modifications of the association between NO_2_ and five transitions of T2D. 23](#_Toc112353249)

# Details of outcomes

**Type 2 diabetes (T2D)**

Cases of T2D were identified by the International Classification of Disease, Tenth version (ICD-10) code families E11 (fields were 130708 and 130709 in UK Biobank). These fields are first occurrence data of diseases, gathering information from self-reported outcomes, primary care, hospital admissions, and death records, and converting them to three-digit ICD-10 categories. We also collected the cases directly from hospital admissions and death records to obtain the latest information.

To exclude the prevalent diabetes cases, we also collected cases of diabetes identified through HbA1c ≥48mmol/mol (field 30750), ICD-9 code 250 in hospital records, algorithmically defined outcome (field 2976), and participants who ever used insulin or other hypoglycemic drugs before recruitment(field 20003).

**Diabetes complication**

Diabetes complication include diabetic eye diseases, diabetic kidney diseases, diabetic neuropathy diseases, cardiovascular diseases, peripheral vascular diseases, and metabolic events.

**Diabetic eye diseases**

Diabetic eye diseases were identified through ICD-10 codes E11.3 (type 2 diabetes mellitus with ophthalmic complications), H36.0 (diabetic retinopathy), and H28.0 (diabetic cataract) in hospital or death records, and corresponding primary care codes.

The prevalent cases were also identified through self-report outcomes (diabetic eye disease; fields 20002, 20008, and 20009) and eye problems (field 6148).

**Diabetic kidney diseases**

Diabetic kidney diseases were identified through ICD-10 codes E11.2 (type 2 diabetes mellitus with renal complications), E18.0 (end-stage renal disease), E18.3-5 (chronic kidney disease stage 3-5), N08.3 (glomerular disorders in diabetes mellitus) in hospital or death records, at least two consecutive eGFR <60 mL/min/1.73m^2^ measured more than 90 days, macroalbuminuria, and microalbuminuria. eGFR was calculated using creatinine measured in UK Biobank accessment center (field 30700) or primary care data. Macroalbuminuria was identified through urine albumin:creatinine ratio (uACR) above 33.9 using fields 30510 and 30500. uACR above 3.4 was considered microalbuminuria.

**Diabetic neuropathy diseases**

Diabetic neuropathy diseases were identified through ICD-10 codes E11.4 (type 2 diabetes mellitus with neurological complications) and G99.0 (autonomic neuropathy in endocrine and metabolic diseases) in hospital or death records, as well as a set of primary care codes.

The prevalent cases were also identified through self-report outcomes (diabetic neuropathy/ulcers).

**Cardiovascular diseases**

Cardiovascular diseases were identified through ICD-10 codes I21 (acute myocardial infarction), I22 (subsequent myocardial infarction), I23 (complications of myocardial infarction), I63 (cerebral infarction), and I64 (stroke) using first occurrence data (field 131298, 131300, 131302, 131366, and 131368), or I20.0 (unstable angina) in hospital or death records, and primary care recodes as well, or OPSC4 codes K40, K41, K42, K43, K44, K45, K46, K483, K49, K501, K75, and K76 (percutaneous coronary intervention). We also obtained algorithmically defined stroke (fields 42006 and 42008), myocardial infarction (field 42000), and self-reported coronary angioplasty (field 1070) and coronary bypass grafts (field 1095).

The prevalent cases were also identified through algorithmically defined outcomes (heart attack, angina, and stroke; field 6150) and self-reported outcome (heart attack and stroke).

**Peripheral vascular diseases**

peripheral vascular diseases were identified through ICD-10 codes E11.5 (type 2 diabetes mellitus with peripheral circulatory complications), I73.8 (other specified peripheral vascular diseases), I73.9 (unspecified peripheral vascular diseases) in hospital or death records, and primary care recodes.

**Metabolic events**

Metabolic events were identified ICD-10 codes E11.0 (type 2 diabetes mellitus with coma), E11.1 (type 2 diabetes mellitus with ketoacidosis), E11.6 (type 2 diabetes mellitus with specified complications) in hospital or death records, and a set of primary care codes.

Other events were identified ICD-10 code E11.8 (type 2 diabetes mellitus with unspecified complications) in hospital or death records, and a set of primary care codes.

**All-cause death**

Death recodes were acquired from central registry and mapped into four-digit ICD-10 code.

**Cause-specific mortality**

We use primary cause of death to identify the cause-specific mortality according to the ICD-10 codes. Cancer mortality was identified based on codes C00-C99. Cardiovascular disease (CVD) mortality was identified based on codes I00-I99. Respiratory disease mortality was identified based on codes J00-J99.

# Table S1. The UDI, definition and measurements of covariates.

| **Variables** | **UDI** | **Unit** | **Notes** |
| --- | --- | --- | --- |
| Age | 21022-0.0 | years | The age referred to the age of the participant on the day they attended an Initial Assessment Centre, truncated to whole year. |
| Sex | 31-0.0 |  | Sex acquired from central registry at recruitment, but in some cases updated by the participant. |
| Ethnicity | 21000-0.0 |  | Ethnicity is an amalgam of sequential branching questions asked during the initial Assessment Centre visit as part of the touchscreen questionnaire.  Reference: white. |
| Residential area | 20118-0.0 |  | The classification is derived by combining each participant’s home postcode with data generated from the 2001 census from the Office of National Statistics, using the Geoconvert tool from Census Dissemination Unit.  Reference: rural. |
| Smoking status | 20116-0.0 |  | The current/past smoking status of the participant.  Reference: never. |
| Healthy diet | 1349-0.0/ 1369-0.0/ 1379-0.0/ 1389-0.0/ 1329-0.0/ 1339-0.0/ 1458-0.0/1289-0.0/  1299-0.0/1309-0.0/1319-0.0/1568-0.0/1578-0.0/1588-0.0/1598-0.0/1608-0.0 |  | Red meat intake  Red meat included beef, lamb, mutton, pork, and processed meat, and it was calculated the sum of them.  Fish intake  Fish included oily fish and non-oily fish, and fish intake was calculated by the sum of them.  Vegetable intake  Vegetable intake was measured with screening questions, and it was calculated as the sum of cooked vegetable intake and raw vegetable intake.  Fruit intake  Fruit intake was measured with screening questions, and it was calculated as the sum of fresh fruit intake and dried fruit intake.  Alcohol consumption  Alcohol consumption was calculated by the quantity of each type of drink (red wine, white wine, beer, cider, fortified wine and spirits), multiplied by its standard drink size and reference alcohol content (Alcohol intake (g/week).  Reference alcohol content (according to website: https://www.drinkaware.co.uk/facts/alcoholic-drinks-and-units/what-is-an-alcohol-unit): 1 unit-equivalent described as containing 8g of pure alcohol; 175ml wine=2.3 units-equivalents, 1 pint beer =2.3 units-equivalents, 25ml spirits=1 unit-equivalent, 62.5ml fortified wine=1 unit-equivalent.  Healthy diet score was evaluated by red meat intake (<median), fish intake (≥median), vegetable intake (≥median), fruit intake (≥median), and alcohol consumption (male <28g/day; female <14g/day).  One point was given for each favorable diet factor and the total diet score ranges from 0 to 5; a healthy diet was defined as a diet score ≥ 3].  Reference: diet score < 3. |
| Physical activity | 22032-0.0 |  | Physical activity was classified according to International Physical Activity Questionnaire (IPAQ).  Classification:  High: 1) vigorous-intensity activity on at least 3 days achieving a minimum Total physical activity of at least 1500 MET-minutes/week; 2) 7 or more days of any combination of walking, moderate-intensity or vigorous-intensity activities achieving a minimum Total physical activity of at least 3000 MET-minutes/week.  Moderate: 1) 3 or more days of vigorous-intensity activity of at least 20 minutes per day; 2) 5 or more days of moderate-intensity activity and/or walking of at least 30 minutes per day; 3) 5 or more days of any combination of walking, moderate-intensity or vigorousintensity activities achieving a minimum Total physical activity of at least 600 MET-minutes/week.  Low: Those individuals who not meet criteria for Categories high or moderate are considered ‘low’.  Reference: low level. |
| Obese | 23104-0.0 | Kg/m^2^ | BMI more than 30kg/m^2^ was defined as obese.  BMI was measured by impedance measurement.  Reference: no obesity. |
| Family history of diabetes | 20107-0.0~3.9/  20110-0.0~3.9/  20111-0.0~3.9 |  | Family history of diabetes was measured with screening questions: “Has/did your mother ever suffer from? (You can select more than one answer)”. Family history included the illness of father, mother, and siblings.  Reference: no family history of diabetes. |
| Hypertension | 93-0.0~0.1/  94-0.0~0.1/  6177-0.0~0.2 |  | Systolic blood pressure and diastolic blood pressure was measured by automated device. The participants were classified as hypertension if their systolic blood pressure >130mmHg or diastolic blood pressure >90mmHg or ever had medication for blood pressure.  Reference: no history of hypertension. |
| High cholesterol | 20002-0.0~0.33/6153-0.0~0.3/6177-0.0~0.3 |  | High cholesterol was defined as a self-reported history of high cholesterol or taking medications.  Reference: no history of high cholesterol. |
| Cancer | 2453-0.0  20001-0.0~0.5  40006-0.0~16.0 |  | Cancer was extracted from the screening question, self-reported data, and national cancer registries.  Reference: no history of cancer. |
| Traffic noise | 24024-0.0 | dB | Traffic noise is a weighted Leq noise level measured over the 24-hour period with a 10 decibel penality added to the levels between 23:00 and 07:00. |

# Table S2. Characteristics of the participants included or excluded in the study.

|  | Excluded  (*n* = 103,468) | Included  (*n* = 398,993) |
| --- | --- | --- |
| Age [years, mean (SD)] | 60.52 (6.89) | 55.49 (8.06) |
| Sex (%) |  |  |
| Male | 58,053 (56.1) | 171,055 (42.9) |
| Female | 45,415 (43.9) | 227,938 (57.1) |
| Ethnicity (%) |  |  |
| White | 95,914 (92.7) | 376,743 (94.4) |
| Non-white | 7554 (7.3) | 22,250 (5.6) |
| Residential area (%) |  |  |
| Urban | 89,247 (86.3) | 343,011 (86.0) |
| Rural | 13,236 (12.8) | 55,982 (14.0) |
| Smoking status (%) |  |  |
| Never | 47,020 (45.4) | 227,700 (57.1) |
| Previous | 42,530 (41.1) | 131,114 (32.9) |
| Current | 13,024 (12.6) | 40,179 (10.1) |
| Obese (%) |  |  |
| Yes | 38,054 (36.8) | 110,765 (27.8) |
| No | 64,405 (62.2) | 387,917 (97.2) |
| Physical activity (%) |  |  |
| Low | 18,205 (17.6) | 72,398 (18.1) |
| Moderate | 31,962 (30.9) | 164,071 (41.1) |
| High | 29,650 (28.7) | 162,524 (40.7) |
| Healthy diet (%) |  |  |
| Yes | 74,428 (71.9) | 284,828 (71.4) |
| No | 28,385 (27.4) | 114,165 (28.6) |
| Chronic diseases (%) |  |  |
| High cholesterol | 51,802 (50.1) | 41,841 (10.5) |
| Hypertension | 71,943 (69.5) | 161,003 (40.4) |
| Cancer | 14,026 (13.6) | 42,225 (10.6) |

# Table S3. Distributions of the annual average exposures among 398,993 participants.

| **Exposure (****μg/m^3^)** | **Mean** | **Standard deviation** | **25th percentile** | **Median** | **75th percentile** | **Interquartile range** | **Minimum** | **Maximum** |
| --- | --- | --- | --- | --- | --- | --- | --- | --- |
| **The whole stage** | | | | | | | | |
| PM_10_ | 15.04 | 2.41 | 13.23 | 14.98 | 16.48 | 3.25 | 2.92 | 28.48 |
| PM_2.5_ | 10.01 | 1.74 | 8.74 | 9.98 | 11.05 | 2.31 | 1.69 | 18.70 |
| NO_x_ | 27.27 | 10.92 | 19.79 | 25.81 | 32.22 | 12.43 | 3.75 | 111.01 |
| NO_2_ | 18.23 | 5.92 | 14.17 | 17.71 | 21.25 | 7.08 | 2.86 | 57.92 |
| **Baseline → T2D** | | | | | | | | |
| PM_10_ | 15.28 | 2.44 | 13.46 | 15.23 | 16.73 | 3.27 | 3.67 | 28.27 |
| PM_2.5_ | 10.19 | 1.75 | 8.93 | 10.18 | 11.26 | 2.33 | 2.21 | 18.86 |
| NO_x_ | 27.88 | 11.09 | 20.24 | 26.39 | 32.99 | 12.75 | 4.31 | 110.99 |
| NO_2_ | 18.59 | 5.97 | 14.52 | 18.06 | 21.63 | 7.11 | 3.29 | 57.60 |
| **T2D → Complication** | | | | | | | | |
| PM_10_ | 15.66 | 2.41 | 13.91 | 15.52 | 17.04 | 3.13 | 8.85 | 25.22 |
| PM_2.5_ | 10.46 | 1.73 | 9.25 | 10.40 | 11.46 | 2.21 | 5.74 | 17.35 |
| NO_x_ | 30.04 | 11.43 | 22.18 | 28.5 | 35.56 | 13.38 | 7.09 | 105.14 |
| NO_2_ | 19.79 | 6.04 | 15.71 | 19.26 | 22.96 | 7.25 | 5.45 | 54.21 |
| **Baseline → Death** | | | | | | | | |
| PM_10_ | 15.25 | 2.43 | 13.44 | 15.20 | 16.71 | 3.27 | 3.67 | 28.27 |
| PM_2.5_ | 10.17 | 1.74 | 8.91 | 10.16 | 11.23 | 2.32 | 2.21 | 18.86 |
| NO_x_ | 27.79 | 11.04 | 20.21 | 26.32 | 32.87 | 12.66 | 4.31 | 110.99 |
| NO_2_ | 18.53 | 5.94 | 14.49 | 18.02 | 21.57 | 7.09 | 3.29 | 57.60 |
| **T2D → Death** | | | | | | | | |
| PM_10_ | 15.40 | 2.4 | 13.71 | 15.30 | 16.78 | 3.07 | 8.85 | 25.22 |
| PM_2.5_ | 10.28 | 1.72 | 9.12 | 10.25 | 11.26 | 2.14 | 5.74 | 17.35 |
| NO_x_ | 29.27 | 11.21 | 21.62 | 27.72 | 34.70 | 13.08 | 7.03 | 105.14 |
| NO_2_ | 19.35 | 5.96 | 15.33 | 18.82 | 22.50 | 7.17 | 5.41 | 54.21 |
| **Complication → Death** | | | | | | | | |
| PM_10_ | 14.91 | 2.41 | 13.15 | 14.85 | 16.36 | 3.21 | 8.93 | 24.29 |
| PM_2.5_ | 9.91 | 1.75 | 8.61 | 9.92 | 10.97 | 2.36 | 5.78 | 16.51 |
| NO_x_ | 28.11 | 10.44 | 20.78 | 26.88 | 33.32 | 12.54 | 7.03 | 100.30 |
| NO_2_ | 18.75 | 5.60 | 14.88 | 18.42 | 21.84 | 6.96 | 5.41 | 51.65 |

Abbreviations: T2D, type 2 diabetes.

# Table S4. Associations between air pollution and risk of cause-specific mortality.

|  | **Cases** | **PM_10_** | **PM_2.5_** | **NO_x_** | **NO_2_** |
| --- | --- | --- | --- | --- | --- |
| Baseline to Diabetes | 13,393 | 1.66 (1.62, 1.69) | 1.63 (1.59, 1.67) | 1.39 (1.37, 1.42) | 1.49 (1.46, 1.51) |
| Diabetes to Complication | 3791 | 1.13 (1.08, 1.18) | 1.08 (1.03, 1.13) | 1.10 (1.06, 1.15) | 1.15 (1.11, 1.20) |
| Baseline to Death |  |  |  |  |  |
| To cancer mortality | 10,966 | 1.52 (1.48, 1.56) | 1.50 (1.46, 1.54) | 1.30 (1.28, 1.33) | 1.37 (1.34, 1.40) |
| To CVD mortality | 1728 | 1.58 (1.49, 1.68) | 1.57 (1.47, 1.67) | 1.35 (1.29, 1.42) | 1.43 (1.36, 1.51) |
| To respiratory mortality | 1061 | 1.51 (1.40, 1.63) | 1.51 (1.40, 1.64) | 1.40 (1.32, 1.48) | 1.48 (1.39, 1.58) |
| T2D to Death |  |  |  |  |  |
| To cancer mortality | 562 | 1.45 (1.30, 1.63) | 1.55 (1.38, 1.74) | 1.20 (1.09, 1.31) | 1.26 (1.13, 1.39) |
| To CVD mortality | 84 | 1.36 (1.01, 1.84) | 1.44 (1.06, 1.95) | 1.32 (1.06, 1.64) | 1.38 (1.08, 1.77) |
| To respiratory mortality | 74 | 1.14 (0.82, 1.58) | 1.19 (0.86, 1.65) | 1.18 (0.91, 1.51) | 1.22 (0.93, 1.61) |
| Complication to Death |  |  |  |  |  |
| To cancer mortality | 176 | 1.44 (1.17, 1.76) | 1.50 (1.22, 1.84) | 1.33 (1.11, 1.59) | 1.40 (1.15, 1.69) |
| To CVD mortality | 102 | 1.69 (1.31, 2.17) | 1.82 (1.41, 2.35) | 1.53 (1.25, 1.87) | 1.62 (1.29, 2.02) |
| To respiratory mortality | 41 | 1.38 (0.93, 2.04) | 1.44 (0.97, 2.14) | 1.44 (1.06, 1.95) | 1.51 (1.07, 2.12) |

HRs (95% CI) are results for per IQR increase in each pollutant. IQR increase was 3.25 μg/m^3^ for PM_10_, 2.31 μg/m^3^ for PM_2.5_, 12.43 μg/m^3^ for NO_x_, and 7.08 μg/m^3^ for NO_2_.

The multivariable model was adjusted for age, sex, ethnicity, residential area, smoking status, healthy diet, physical activity, and family history of diabetes.

Abbreviations: CI, confidence interval; CVD, cardiovascular diseases; HR, hazard ratio; IQR, interquartile range; T2D, type 2 diabetes.

# Table S5. Results of 2-pollutant models.

| Pollutant | model | Baseline → T2D | T2D → Complication | Baseline → Death | T2D → Death | Complication → Death | *p* for heterogeneity |
| --- | --- | --- | --- | --- | --- | --- | --- |
| PM_10_ | + NO_x_ | 1.67 (1.61, 1.74) | 1.07 (0.99, 1.16) | 1.54 (1.49, 1.60) | 1.56 (1.33, 1.82) | 1.33 (1.06, 1.67) | 0.152 |
| PM_10_ | + NO_2_ | 1.53 (1.47, 1.59) | 0.97 (0.89, 1.05) | 1.43 (1.38, 1.48) | 1.45 (1.23, 1.69) | 1.27 (1.01, 1.60) | <0.001 |
| PM_2.5_ | + NO_x_ | 1.52 (1.46, 1.58) | 0.95 (0.87, 1.02) | 1.46 (1.41, 1.51) | 1.82 (1.55, 2.14) | 1.54 (1.23, 1.92) | <0.001 |
| PM_2.5_ | + NO_2_ | 1.40 (1.34, 1.46) | 0.85 (0.79, 0.92) | 1.36 (1.32, 1.41) | 1.70 (1.45, 1.99) | 1.48 (1.18, 1.85) | <0.001 |
| NO_x_ | + PM_10_ | 0.99 (0.96, 1.02) | 1.05 (0.99, 1.13) | 0.98 (0.95, 1.01) | 0.91 (0.80, 1.03) | 1.11 (0.91, 1.35) | <0.001 |
| NO_x_ | + PM_2.5_ | 1.06 (1.03, 1.10) | 1.15 (1.08, 1.22) | 1.02 (1.00, 1.05) | 0.83 (0.73, 0.94) | 1.00 (0.83, 1.22) | <0.001 |
| NO_2_ | + PM_10_ | 1.08 (1.05, 1.12) | 1.18 (1.10, 1.27) | 1.06 (1.03, 1.09) | 0.97 (0.85, 1.12) | 1.17 (0.95, 1.46) | <0.001 |
| NO_2_ | + PM_2.5_ | 1.17 (1.13, 1.21) | 1.30 (1.21, 1.39) | 1.11 (1.07, 1.14) | 0.88 (0.76, 1.00) | 1.05 (0.85, 1.29) | <0.001 |

HRs (95% CI) are results for per IQR increase in each pollutant. IQR increase was 3.25 μg/m^3^ for PM_10_, 2.31 μg/m^3^ for PM_2.5_, 12.43 μg/m^3^ for NO_x_, and 7.08 μg/m^3^ for NO_2_.

*p* for heterogeneity was estimated by comparing the nested single- and 2-pollutant models by likelihood ratio tests.

The multivariable model was additionally adjusted for age, sex, ethnicity, residential area, smoking status, healthy diet, physical activity, and family history of diabetes.

Abbreviations: CI, confidence interval; HR, hazard ratio; T2D, type 2 diabetes.

# Table S6. Results of sensitivity analyses in the model including traffic noise (*n* = 393,515).

| Transition | Cases | PM_10_ | PM_2.5_ | NO_x_ | NO_2_ |
| --- | --- | --- | --- | --- | --- |
| Baseline → T2D | 13,209 | 1.67 (1.63, 1.71) | 1.64 (1.60, 1.68) | 1.41 (1.38, 1.43) | 1.50 (1.47, 1.53) |
| T2D → Complication | 3715 | 1.15 (1.10, 1.21) | 1.11 (1.06, 1.16) | 1.12 (1.08, 1.17) | 1.18 (1.13, 1.23) |
| Baseline → Death | 17,213 | 1.52 (1.49, 1.55) | 1.51 (1.48, 1.54) | 1.32 (1.30, 1.34) | 1.39 (1.37, 1.42) |
| T2D → Death | 909 | 1.43 (1.31, 1.57) | 1.52 (1.38, 1.67) | 1.23 (1.14, 1.33) | 1.29 (1.19, 1.40) |
| Complication → Death | 405 | 1.45 (1.26, 1.66) | 1.53 (1.33, 1.75) | 1.35 (1.20, 1.52) | 1.41 (1.24, 1.60) |

HRs (95% CI) are results for per IQR increase in each pollutant. IQR increase was 3.23 μg/m^3^ for PM_10_, 2.30 μg/m^3^ for PM_2.5_, 12.39 μg/m^3^ for NO_x_, and 7.05 μg/m^3^ for NO_2_.

The multivariable model was adjusted for age, sex, ethnicity, residential area, smoking status, healthy diet, physical activity, family history of diabetes, and traffic noise.

Abbreviations: CI, confidence interval; HR, hazard ratio; T2D, type 2 diabetes.

# Table S7. Results of sensitivity analyses using different time intervals.

| Transition | Cases | PM_10_ | PM_2.5_ | NO_x_ | NO_2_ |
| --- | --- | --- | --- | --- | --- |
| **1 day interval (*n* = 398,993)** | | | | | |
| Baseline → T2D | 13,393 | 1.63 (1.59, 1.67) | 1.62 (1.59, 1.66) | 1.39 (1.37, 1.41) | 1.48 (1.45, 1.51) |
| T2D → Complication | 3791 | 1.11 (1.06, 1.16) | 1.05 (1.00, 1.10) | 1.10 (1.06, 1.14) | 1.15 (1.11, 1.20) |
| Baseline → Death | 17,510 | 1.51 (1.48, 1.54) | 1.50 (1.47, 1.53) | 1.32 (1.30, 1.34) | 1.39 (1.36, 1.41) |
| T2D → Death | 924 | 1.37 (1.25, 1.50) | 1.45 (1.32, 1.59) | 1.20 (1.11, 1.29) | 1.25 (1.16, 1.35) |
| Complication → Death | 411 | 1.46 (1.28, 1.67) | 1.54 (1.35, 1.76) | 1.36 (1.22, 1.52) | 1.42 (1.25, 1.60) |
| **1 year interval (*n* = 398,993)** | | | | | |
| Baseline → T2D | 13,393 | 1.64 (1.61, 1.68) | 1.63 (1.59, 1.66) | 1.39 (1.37, 1.42) | 1.48 (1.46, 1.51) |
| T2D → Complication | 3791 | 1.12 (1.07, 1.17) | 1.07 (1.02, 1.12) | 1.10 (1.06, 1.14) | 1.15 (1.11, 1.20) |
| Baseline → Death | 17,510 | 1.51 (1.48, 1.54) | 1.50 (1.47, 1.53) | 1.32 (1.30, 1.34) | 1.39 (1.36, 1.41) |
| T2D → Death | 924 | 1.39 (1.27, 1.53) | 1.48 (1.35, 1.62) | 1.21 (1.13, 1.30) | 1.26 (1.17, 1.37) |
| Complication → Death | 411 | 1.46 (1.28, 1.67) | 1.54 (1.35, 1.76) | 1.36 (1.22, 1.52) | 1.42 (1.25, 1.60) |
| **3 years interval (*n* = 398,796)** | | | | | |
| Baseline → T2D | 13,155 | 1.68 (1.64, 1.72) | 1.65 (1.61, 1.68) | 1.40 (1.38, 1.43) | 1.50 (1.47, 1.52) |
| T2D → Complication | 3553 | 1.15 (1.09, 1.20) | 1.12 (1.07, 1.17) | 1.12 (1.07, 1.16) | 1.16 (1.12, 1.21) |
| Baseline → Death | 17,510 | 1.51 (1.48, 1.54) | 1.50 (1.47, 1.53) | 1.32 (1.30, 1.34) | 1.39 (1.36, 1.41) |
| T2D → Death | 924 | 1.43 (1.31, 1.56) | 1.52 (1.38, 1.66) | 1.22 (1.14, 1.32) | 1.28 (1.18, 1.38) |
| Complication → Death | 385 | 1.41 (1.23, 1.61) | 1.49 (1.30, 1.71) | 1.32 (1.17, 1.48) | 1.37 (1.21, 1.56) |

HRs (95% CI) are results for per IQR increase from multi-state models. IQR increase was 3.25 μg/m^3^ for PM_10_, 2.31 μg/m^3^ for PM_2.5_, 12.43 μg/m^3^ for NO_x_, and 7.08 μg/m^3^ for NO_2_.

The multivariable model was adjusted for age, sex, ethnicity, residential area, smoking status, healthy diet, physical activity, and family history of diabetes.

Abbreviations: CI, confidence interval; HR, hazard ratio; T2D, type 2 diabetes.

# Table S8. Results of sensitivity analyses after excluding deaths not from diabetes or diabetes complication (*n* = 380,437).

| Transition | Cases | PM_10_ | PM_2.5_ | NO_x_ | NO_2_ |
| --- | --- | --- | --- | --- | --- |
| Baseline → T2D | 12,347 | 1.66 (1.62, 1.70) | 1.64 (1.60, 1.68) | 1.40 (1.37, 1.42) | 1.49 (1.46, 1.52) |
| T2D → Complication | 3519 | 1.14 (1.09, 1.19) | 1.09 (1.04, 1.15) | 1.11 (1.07, 1.15) | 1.16 (1.11, 1.21) |
| T2D → Death | 150 | 1.00 (0.78, 1.28) | 1.03 (0.81, 1.32) | 0.99 (0.80, 1.22) | 1.01 (0.81, 1.26) |
| Complication → Death | 139 | 1.39 (1.11, 1.75) | 1.48 (1.18, 1.85) | 1.33 (1.10, 1.61) | 1.37 (1.11, 1.69) |

HRs (95% CI) are results for per IQR increase in each pollutant. IQR increase was 3.24 μg/m^3^ for PM_10_, 2.30 μg/m^3^ for PM_2.5_, 12.35 μg/m^3^ for NO_x_, and 7.02 μg/m^3^ for NO_2_.

The multivariable model was adjusted for age, sex, ethnicity, residential area, smoking status, healthy diet, physical activity, and family history of diabetes.

Abbreviations: CI, confidence interval; HR, hazard ratio; T2D, type 2 diabetes.

# Table S9. Results of sensitivity analyses using complete data (*n* = 318,019).

| Transition | Cases | PM_10_ | PM_2.5_ | NO_x_ | NO_2_ |
| --- | --- | --- | --- | --- | --- |
| Baseline → T2D | 9933 | 1.63 (1.59, 1.67) | 1.60 (1.56, 1.64) | 1.37 (1.34, 1.39) | 1.45 (1.42, 1.48) |
| T2D → Complication | 2831 | 1.12 (1.06, 1.18) | 1.08 (1.02, 1.14) | 1.12 (1.07, 1.17) | 1.17 (1.12, 1.22) |
| Baseline → Death | 13,425 | 1.51 (1.47, 1.54) | 1.49 (1.46, 1.53) | 1.30 (1.28, 1.33) | 1.37 (1.35, 1.40) |
| T2D → Death | 682 | 1.42 (1.28, 1.57) | 1.51 (1.36, 1.68) | 1.21 (1.12, 1.31) | 1.26 (1.16, 1.38) |
| Complication → Death | 290 | 1.56 (1.34, 1.81) | 1.64 (1.41, 1.91) | 1.38 (1.21, 1.56) | 1.44 (1.25, 1.65) |

HRs (95% CI) are results for per IQR increase in each pollutant. IQR increase was 3.28 μg/m^3^ for PM_10_, 2.33 μg/m^3^ for PM_2.5_, 12.36 μg/m^3^ for NO_x_, and 7.02 μg/m^3^ for NO_2_.

The multivariable model was adjusted for age, sex, ethnicity, residential area, smoking status, healthy diet, physical activity, and family history of diabetes.

Abbreviations: CI, confidence interval; HR, hazard ratio; T2D, type 2 diabetes.

# Table S10. Results excluding the participants diagnosed with T2D and complications on the same date (*n* = 396,961).

| Transition | Cases | PM_10_ | PM_2.5_ | NO_x_ | NO_2_ |
| --- | --- | --- | --- | --- | --- |
| Baseline → T2D | 11,361 | 1.76 (1.72, 1.80) | 1.78 (1.74, 1.83) | 1.43 (1.41, 1.46) | 1.53 (1.50, 1.56) |
| T2D → Complication | 1759 | 1.43 (1.35, 1.53) | 1.47 (1.37, 1.56) | 1.25 (1.19, 1.31) | 1.31 (1.24, 1.39) |
| Baseline → Death | 17,510 | 1.51 (1.48, 1.54) | 1.49 (1.47, 1.52) | 1.32 (1.30, 1.34) | 1.39 (1.36, 1.41) |
| T2D → Death | 924 | 1.37 (1.25, 1.50) | 1.45 (1.32, 1.59) | 1.20 (1.11, 1.29) | 1.25 (1.16, 1.35) |
| Complication → Death | 223 | 1.30 (1.08, 1.56) | 1.40 (1.16, 1.70) | 1.23 (1.06, 1.42) | 1.29 (1.09, 1.52) |

HRs (95% CI) are results for per IQR increase in each pollutant. IQR increase was 3.25 μg/m^3^ for PM_10_, 2.31 μg/m^3^ for PM_2.5_, 12.43 μg/m^3^ for NO_x_, and 7.08 μg/m^3^ for NO_2_.

The multivariable model was adjusted for age, sex, ethnicity, residential area, smoking status, healthy diet, physical activity, and family history of diabetes.

Abbreviations: CI, confidence interval; HR, hazard ratio; T2D, type 2 diabetes.


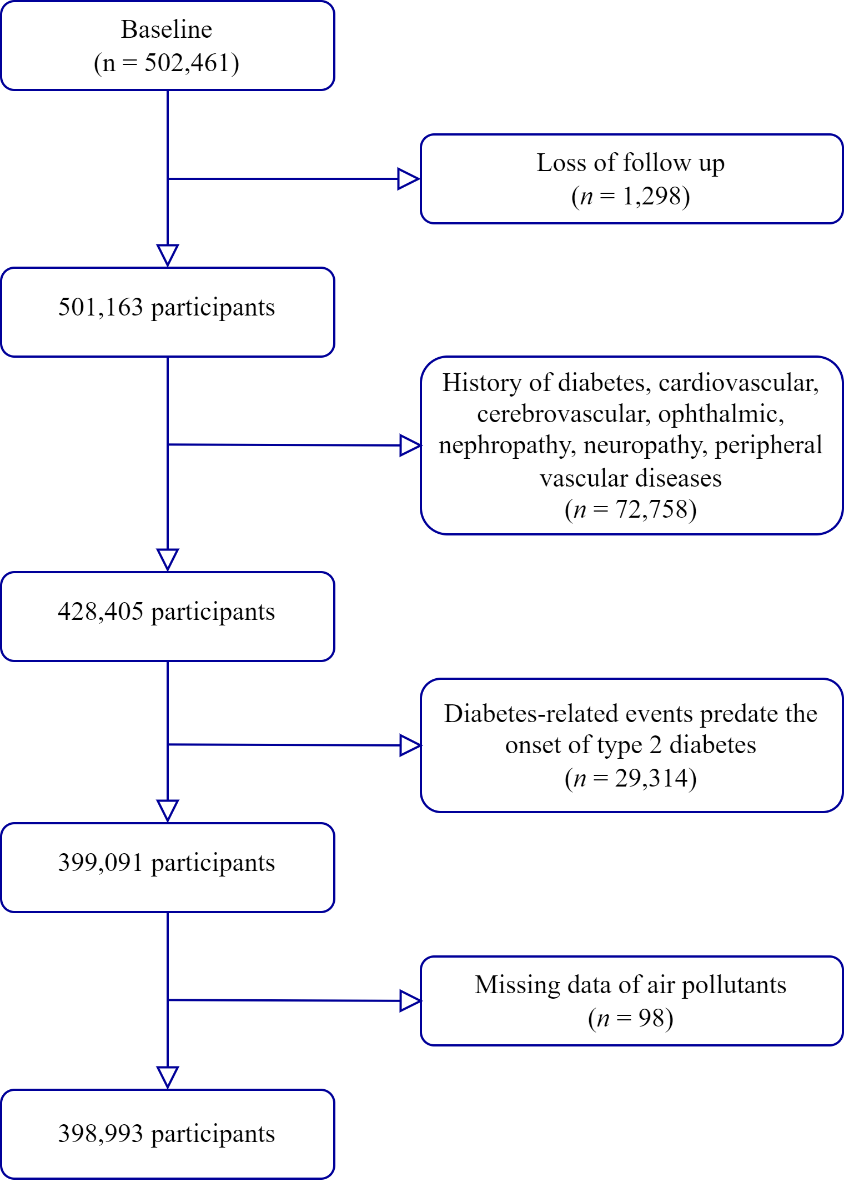


# Figure S1. Flowchart of participants included in this study.

**
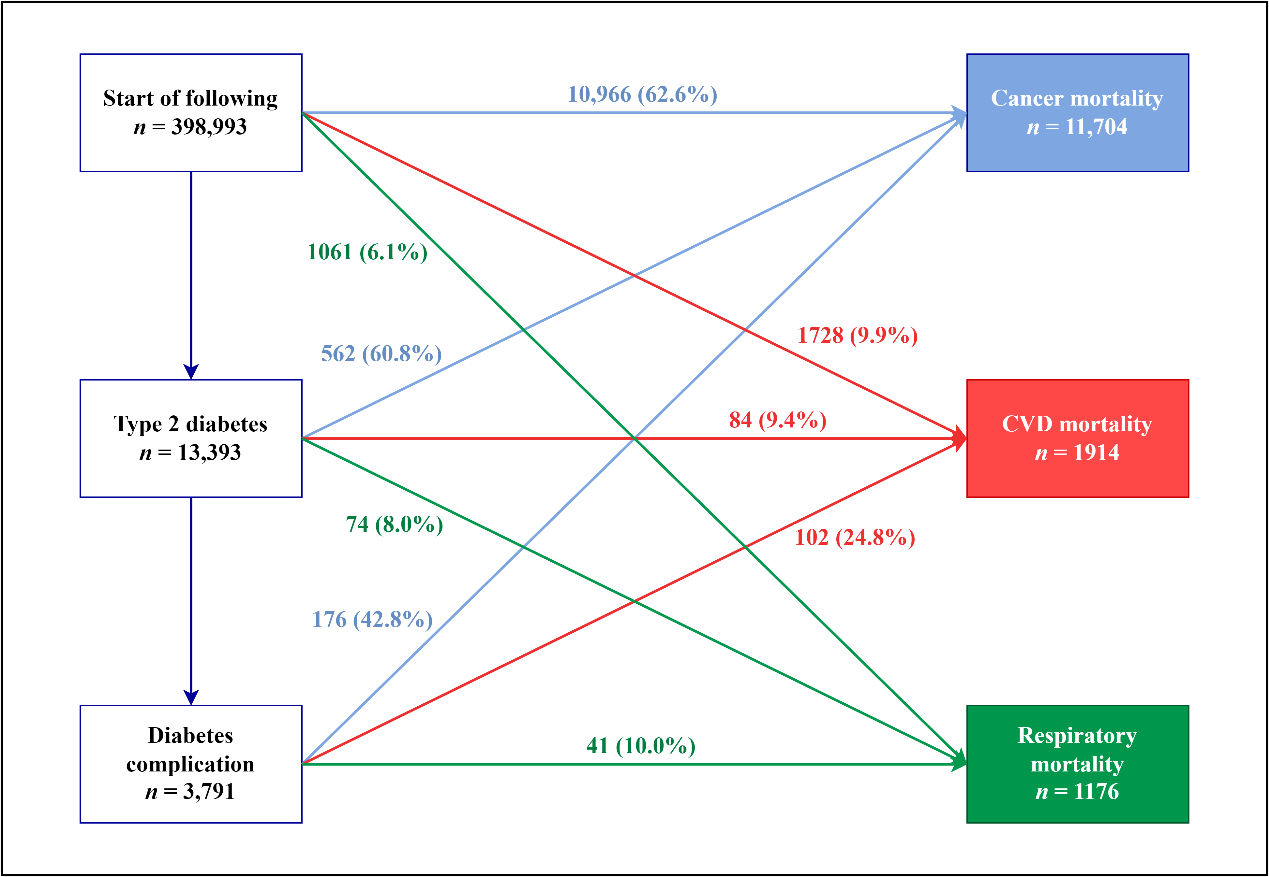
**

# Figure S2. Transitions from baseline to T2D, diabetes complication, and cause-specific mortality.

Cause-specific mortality includes cancer (blue), CVD (red), and respiratory disease mortality (green).

State-specific number of events are reported in boxes, and transition-specific number of events and proportion of mortality in corresponding transition to death (within brackets) are reported on arrows.

Abbreviation: CVD, cardiovascular disease.

**
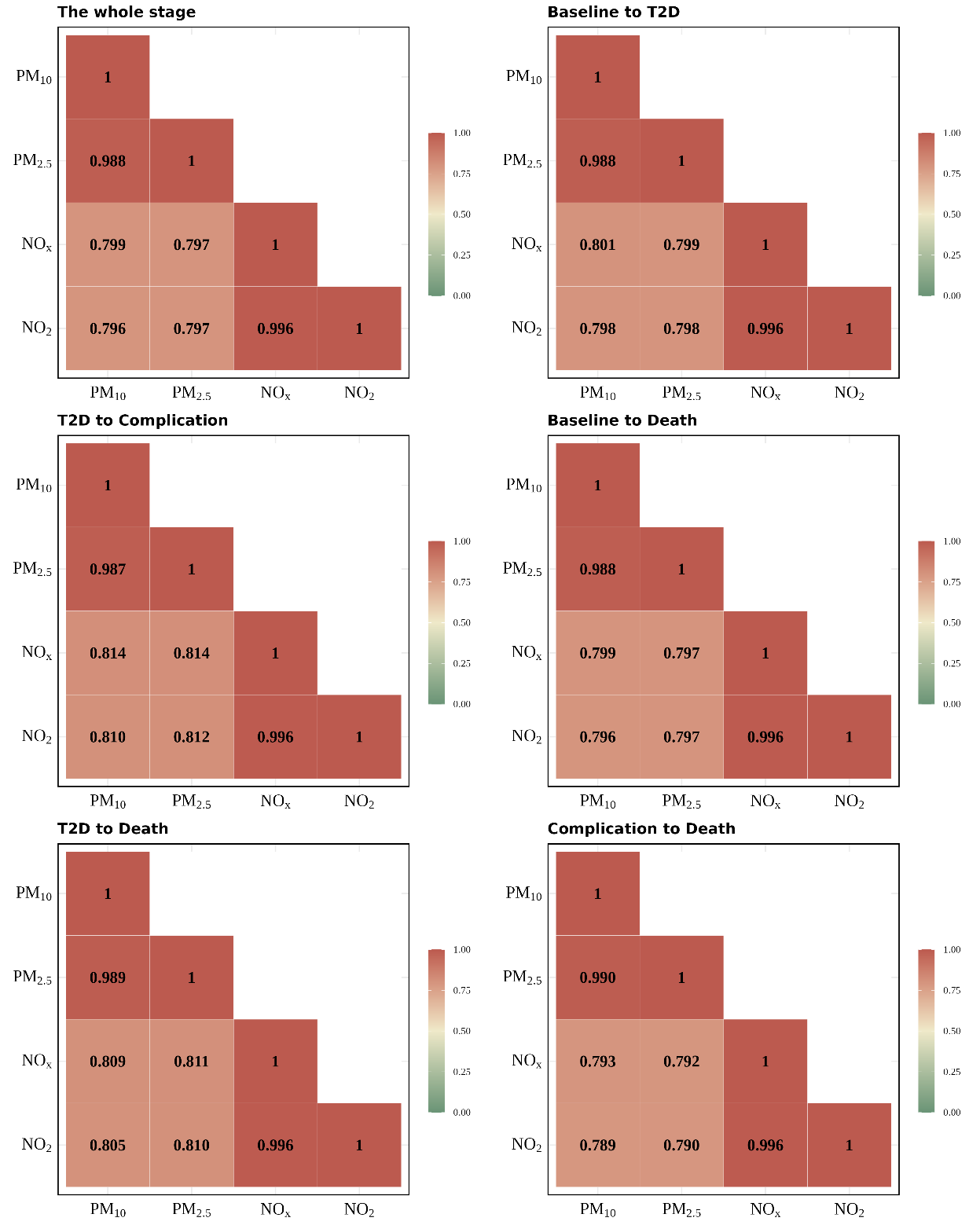
**

# Figure S3. Spearman’s correlation coefficients between air pollutant exposures.

*p* for all pairwise correlations < 0.05.

Abbreviation: T2D: type 2 diabetes.


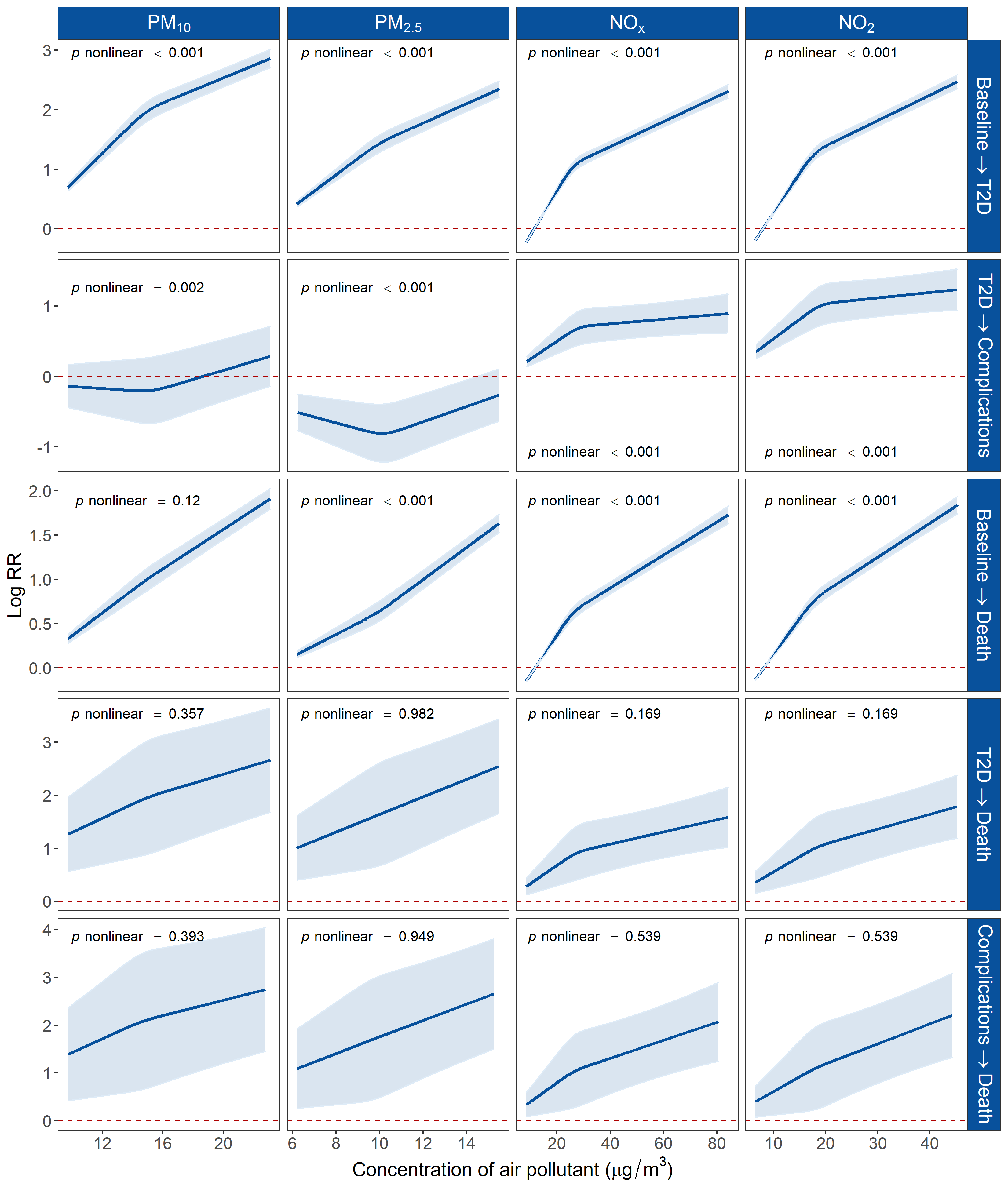


# Figure S4. Exposure-response associations between air pollution exposure and different transitions of T2D.

Associations were modelled by restricted cubic splines with 3 knots. Nonlinear associations were tested using likelihood ratio tests. Model adjusted for age, sex, ethnicity, residential area, smoking status, healthy diet, physical activity, and family history of diabetes.

Abbreviations: RR, relative risk; T2D: type 2 diabetes.


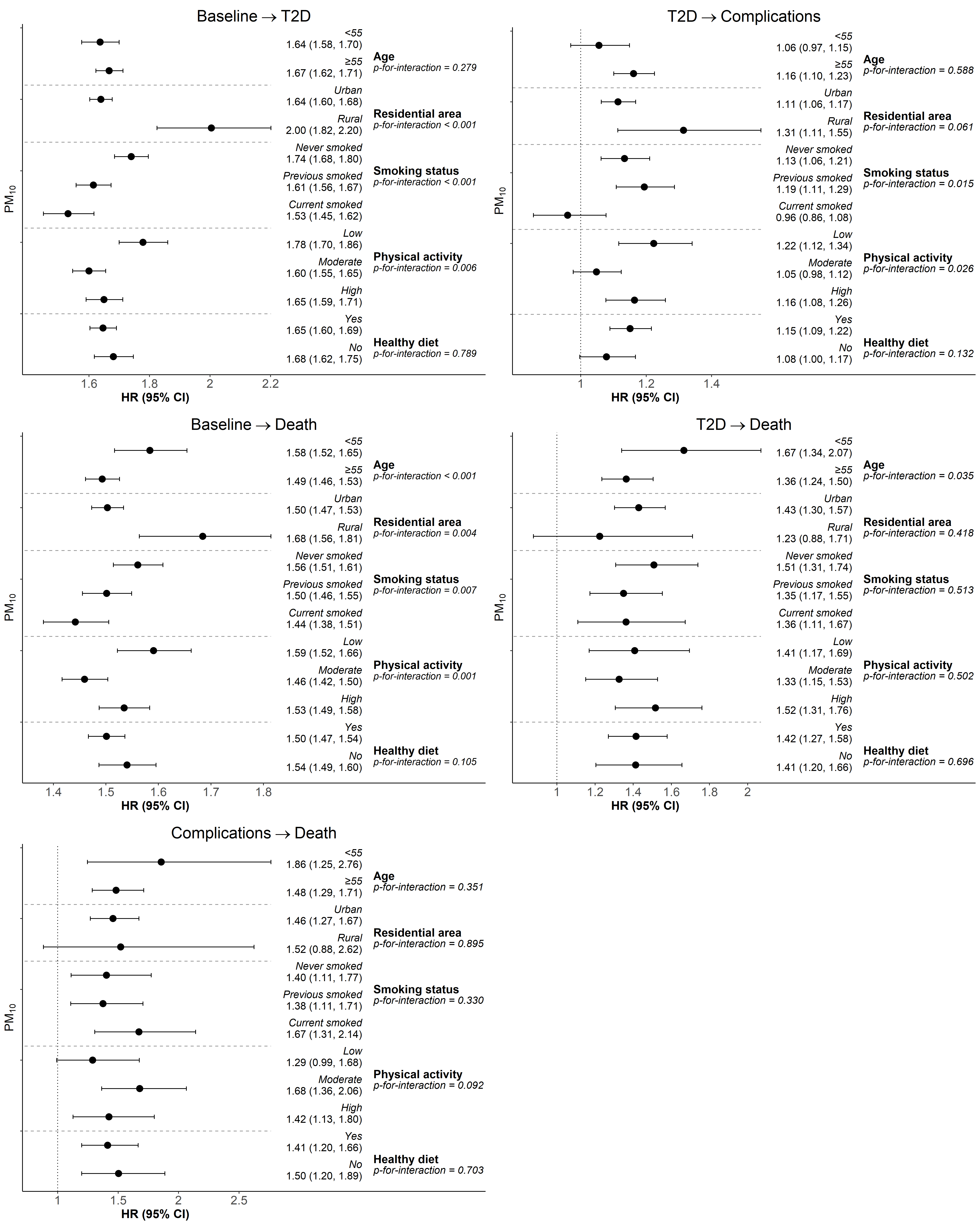


# Figure S5. Effect modifications of the association between PM_10_ and five transitions of T2D.

HRs (95% CI) are results for per IQR increase from multi-state models.

Abbreviations: HR, hazard ratio; T2D: type 2 diabetes.


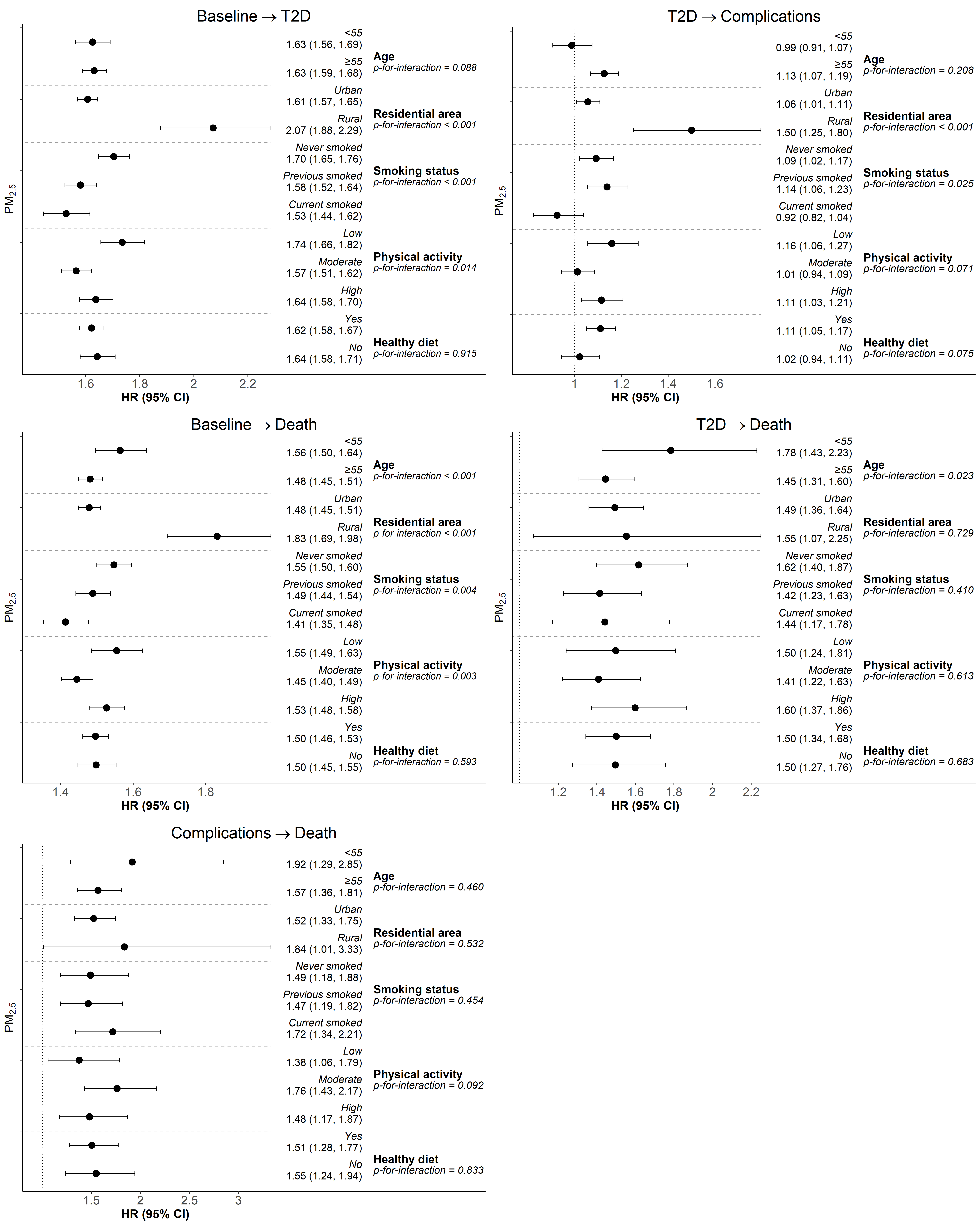


# Figure S6. Effect modifications of the association between PM_2.5_ and five transitions of T2D.

HRs (95% CI) are results for per IQR increase from multi-state models.

Abbreviations: HR, hazard ratio; T2D: type 2 diabetes.


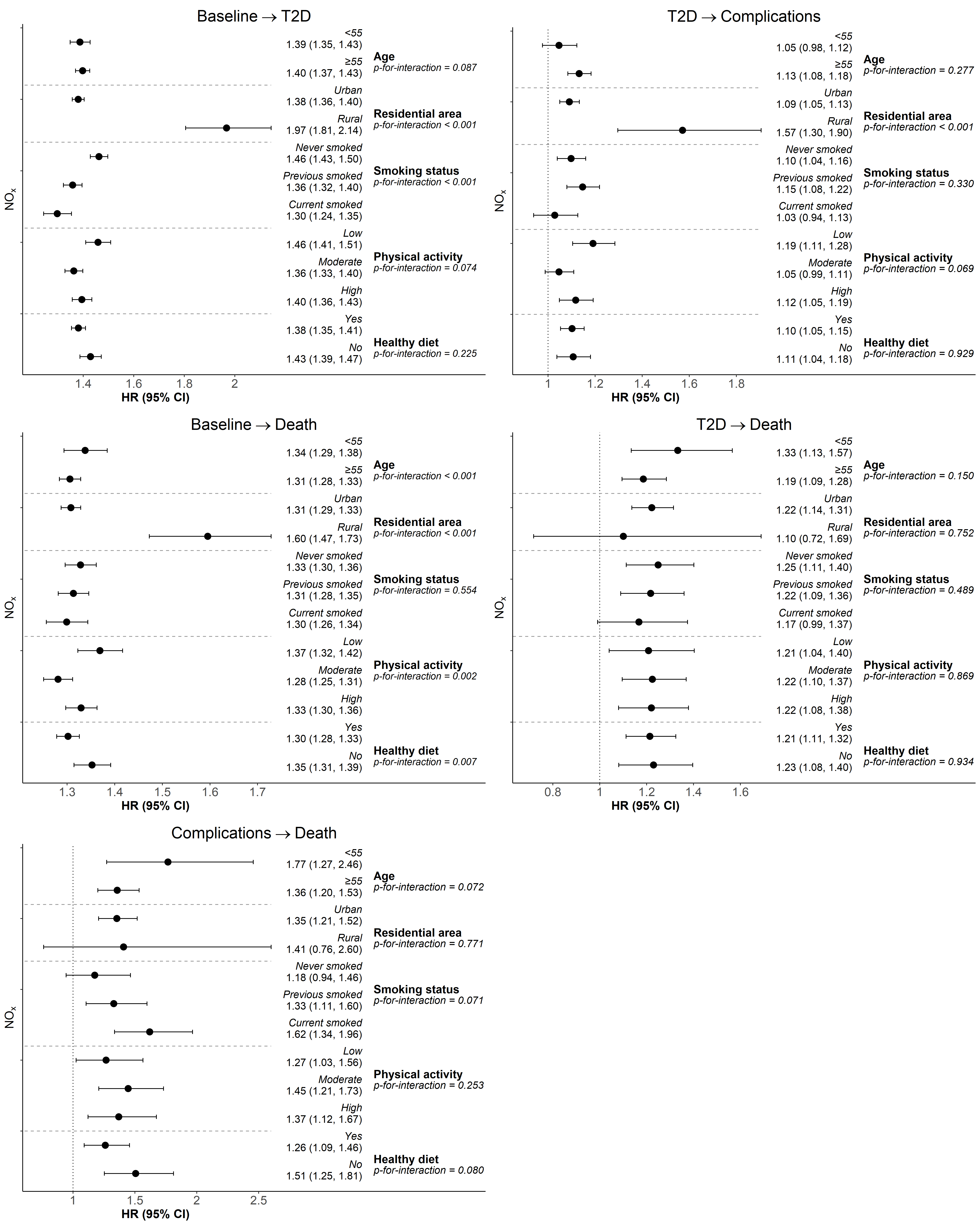


# Figure S7. Effect modifications of the association between NO_x_ and five transitions of T2D.

HRs (95% CI) are results for per IQR increase from multi-state models.

Abbreviations: HR, hazard ratio.


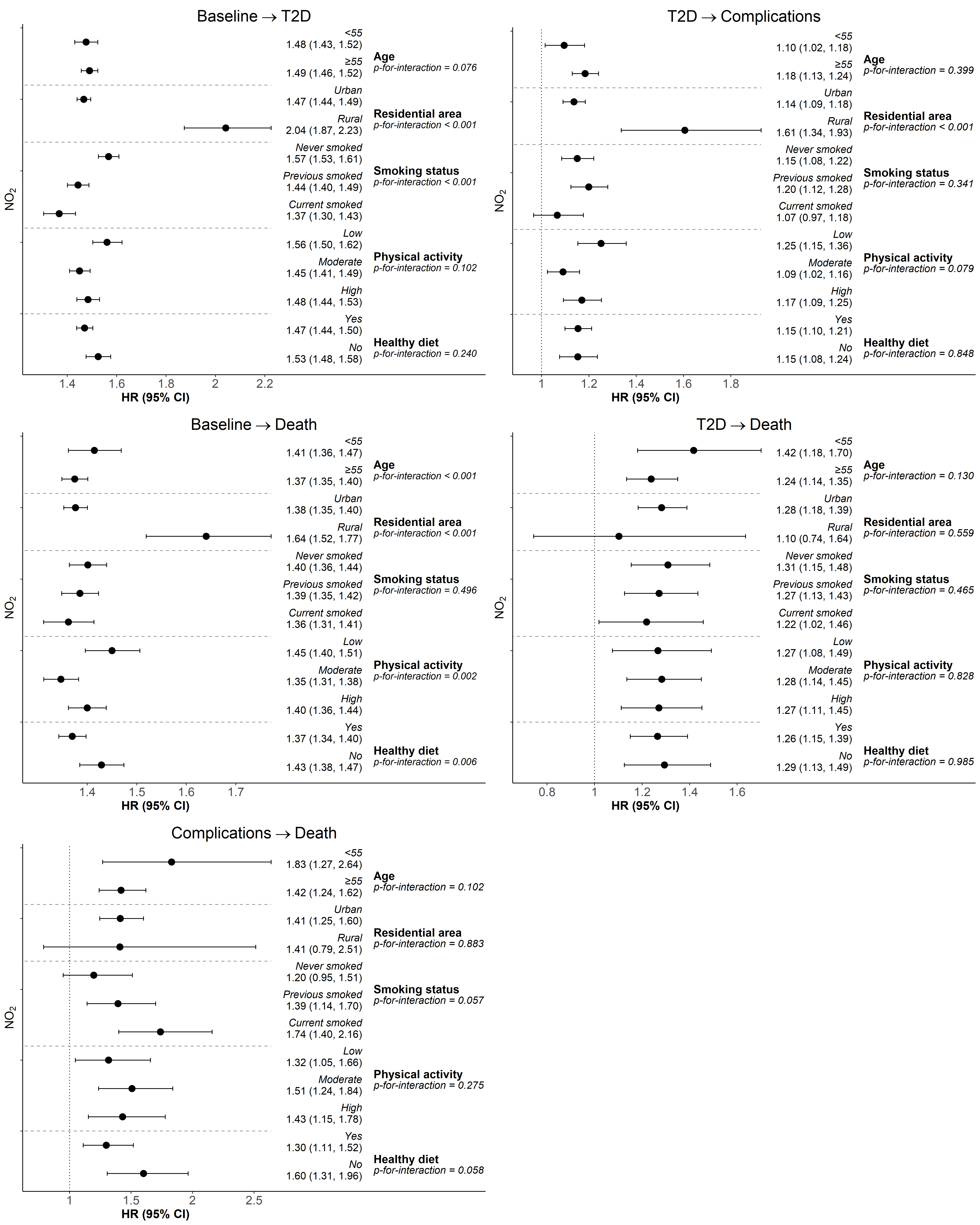


# Figure S8. Effect modifications of the association between NO_2_ and five transitions of T2D.

HRs (95% CI) are results for per IQR increase from multi-state models.

Abbreviations: HR, hazard ratio; T2D: type 2 diabetes.
